# Alfaxalone does not have long‐term effects on goldfish pyramidal neuron action potential properties or GABA_A_ receptor currents

**DOI:** 10.1002/2211-5463.13777

**Published:** 2024-02-11

**Authors:** Domenic Di Stefano, Haushe Suganthan, Leslie Buck

**Affiliations:** ^1^ Department of Cell and Systems Biology University of Toronto Canada; ^2^ Department of Ecology and Evolutionary Biology University of Toronto Canada

**Keywords:** alfaxalone, common goldfish, GABA, GABA‐A receptor, pyramidal neurons

## Abstract

Anesthetics have varying physiological effects, but most notably alter ion channel kinetics. Alfaxalone is a rapid induction and washout neuroactive anesthetic, which potentiates γ‐aminobutyric acid (GABA)‐activated GABA_A_ receptor (GABA_A_‐R) currents. This study aims to identify any long‐term effects of alfaxalone sedation on pyramidal neuron action potential and GABA_A_‐R properties, to determine if its impact on neuronal function can be reversed in a sufficiently short timeframe to allow for same‐day electrophysiological studies in goldfish brain. The goldfish (*Carassius auratus*) is an anoxia‐tolerant vertebrate and is a useful model to study anoxia tolerance mechanisms. The results show that alfaxalone sedation did not significantly impact action potential properties. Additionally, the acute application of alfaxalone onto naive brain slices caused the potentiation of whole‐cell GABA_A_‐R current decay time and area under the curve. Following whole‐animal sedation with alfaxalone, a 3‐h wash of brain slices in alfaxalone‐free saline, with saline exchanged every 30 min, was required to remove any potentiating impact of alfaxalone on GABA_A_‐R whole‐cell currents. These results demonstrate that alfaxalone is an effective anesthetic for same‐day electrophysiological experiments with goldfish brain slices.

## Graphical abstract

Alfaxalone is a rapid induction and washout neuroactive anesthetic, which potentiates γ‐aminobutyric acid (GABA)‐activated GABA_A_ receptor currents. Our study determined that the effect of alfaxalone sedation on goldfish neuronal function is reversed after 3 h. These results demonstrate that alfaxalone is an effective anesthetic for same‐day electrophysiological experiments with goldfish brain slices.

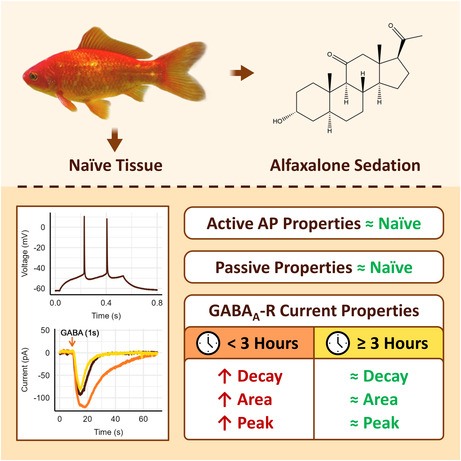

**Abbreviations**
aCSF: artificial cerebrospinal fluid
alfaxalone: 3α‐hydroxy‐5α‐pregnane‐11,20‐dione
AP: action potential
APth: action potential threshold
E_GABA_
: GABA reversal potential
GABA: γ‐aminobutyric acid
GABA_A_‐R: GABA_A_ receptor
h‐PXR: human pregnane X receptors
I/V: current–voltage
LJP: liquid junction potential
MS‐222: tricaine methanesulphonate
SA: spike arrest
TTX: tetrodotoxin

In the early 1970s, the anesthetic alfaxalone (3α‐hydroxy‐5α‐pregnane‐11,20‐dione) was first utilized in humans under the name Althesin, and for veterinary use under the name Saffan [1]. The original formulation for human use—Althesin, was later discontinued for anesthetic use in the mid‐1970s due to multiple reports of anaphylactic reactions in response to one of the components of the drug formulation [2]. In more recent years, a newly formulated neuroactive anesthetic compound—alfaxalone—has been approved for use in Canada.

Alfaxalone is a neuroactive steroid that binds to the γ‐aminobutyric acid (GABA) activated GABA_A_ receptor (GABA_A_‐R) [3] and the anesthetic acts as a potent co‐agonist, potentiating the channel opening effect of GABA through prolonging the mean open time of the GABA_A_‐R [4, 5, 6, 7]. Alfaxalone can induce rapid anesthesia induction, followed by a rapid recovery period. This is supported by the low partition coefficient value, indicating a relatively low lipophilicity, and therefore an increased likelihood to diffuse out of the cellular membrane [8]. Further, the anesthetic induces minimal, cardio‐respiratory depression, especially at lower concentrations; however, these effects have been shown to be dose dependent [9, 10]. Rapid behavioral responses to alfaxalone are associated with low concentrations required to induce sedation, alongside favorable pharmacokinetics, indicating a rapid half‐life, and therefore a rapid washout period in mammals [11, 12, 13, 14, 15].

Alfaxalone has also been shown to successfully induce effective anesthesia in other animal models including fish [16, 17, 18, 19]. However, the analysis of alfaxalone elimination and half‐life times in fish are limited, especially when considering brain tissue. Alfaxalone residue clearance in rainbow trout liver, kidney, heart, spleen, and muscle: however, has been quantified to support a general timeframe of alfaxalone elimination from fish tissue. Following sedation induced with 5 mg·L^−1^ and maintained with 2 mg·L^−1^ alfaxalone, an 84–93% decrease in alfaxalone occurred within the first hour of measurements. Within 2 h of measurements, a reduction of up to 99% of alfaxalone was determined, further illustrating the rapid washout capabilities of the anesthetic [20].

Fish welfare during non‐invasive procedures such as transport and handling, and experimentation involving invasive techniques often require light or full anesthesia [21, 22]. In addition, fish currently represent the second largest group of animals used for research, according to the Canadian Council for Animal Care [23]. The rapid induction and washout of alfaxalone could, therefore, play an important role in improving a variety of invasive fish studies including neuroendocrine, energetics, and anoxia tolerance studies in model organism fish species. A fish species of particular interest in anoxia tolerance studies is one of the most hypoxia‐tolerant vertebrate species, the crucian carp (*Carassius carassius*). Oxygen consumption in this species of fish is able to be maintained at normal levels as low as 5% oxygen air saturation [24], and it can survive complete anoxia for months at temperatures close to 0 °C [25, 26]. Closely related to the crucian carp, is the common goldfish (*Carassius auratus*) which displays similar anoxia tolerance characteristics. The common goldfish has a half‐lethal time of 45 h under anoxic conditions at 5 °C and 22 h at 20 °C and therefore, while not as extremely hypoxia tolerant as the crucian carp, is an inexpensive, abundant, and convenient model organism to help better understand anoxia tolerance mechanisms [25, 27].

In contrast to the extremely anoxia‐tolerant common goldfish, if the human brain is deprived of an adequate source of oxygen for only 5 min, neuronal damage and cell death results [28]. To limit the deleterious excitotoxic cell death which occurs alongside the mammalian response to hypoxia, mechanisms have been identified in anoxia‐tolerant animal models including the common goldfish. The first anoxia‐tolerant mechanism to be proposed was the metabolic arrest hypothesis [29, 30]. Direct calorimetry experiments demonstrated a 70% reduction in heat production, and therefore metabolic rate, during the transition from normoxia to anoxia in the common goldfish [31, 32]. The “ion channel arrest” hypothesis was then proposed as an anoxia tolerance response in turtle brain by Hochachka [33]. Blocking glutamatergic channel activity has been shown to be neuroprotective in mammal hippocampal pyramidal neurons during ischemia [34]. The common goldish experiences a 40–50% decrease in NMDA activity following 40 min of acute anoxia [35]. Contradictory to glutamatergic channel arrest, the identification of a five fold increase in the concentration of the inhibitory neurotransmitter, GABA, would then form the basis for the “spike arrest” (SA) hypothesis [36]. GABA perfusion resulted in the depolarization of pyramidal neuron membrane potential to the GABA reversal potential (E_GABA_) through increased presynaptic GABA_B_‐R activity and postsynaptic GABA_A_‐R channel activity, alongside a decrease in action potential (AP) or “spike” activity [37]. The most recent hypothesis, the “synaptic arrest” hypothesis, combines the increase in inhibitory channel currents and decrease in excitatory channel currents, central to the two previous theories, into one overarching mechanism [38].

In order to further the understanding of anoxia‐tolerant mechanisms utilized by the common goldfish brain, decapitation followed by whole‐brain removal is required to allow for the telencephalon slice preparation used for electrophysiological analysis. However, studies have identified the presence of nociceptors in bony fish, including the common goldfish, similar in both function and structure to mammal pain receptors. It is, therefore, possible that bony fish experience pain during decapitation [39, 40, 41]. While there is still a large debate regarding whether fish experience pain in a manner similar to mammals, fish display nocifensive responses following the application of noxious stimuli, therefore any means to reduce these responses would be beneficial [42, 43, 44]. In addition, goldfish are anoxia tolerant and therefore are likely alert for minutes after decapitation; therefore, it is imperative that a pre‐decapitation anesthetic be found to minimize any possibility for pain and maximize animal well‐being for electrophysiological experimentation. Anesthetic use presents a method of blocking pain perception, commonly used alongside surgical procedures in fish, as long as it doesn't confound electrophysiological experiments [44].

The two most common anesthetic compounds for use in fish include tricaine methanesulphonate (MS‐222) and benzocaine. Following uptake through the gills, both compounds function similarly, through impeding the propagation of action potentials toward the central nervous system. The compounds induce anesthetic effects through the blockage of voltage‐gated sodium channels [41, 45]. Furthermore, MS‐222 has a half‐life of 1.5–4 h in the blood, and remains detectable up to 8 h following administration [46], while benzocaine has an elimination time of up to 25 h [47]. As a result, the use of both compounds can confound electrophysiological measurements [39, 41].

Additional anesthetics include the structurally similar compounds isoeugenol and eugenol or clove oil. Both compounds impede the function of multiple channels involved in the proposed anoxia tolerance pathways. These channels include the NMDA receptor and GABA_A_‐R, which are both key in the synaptic arrest hypothesis, alongside additional sodium, potassium, and calcium channels [41, 48, 49, 50, 51]. Furthermore, eugenol has a half‐life of 12.14 h in the blood [52]. As a result, high concentrations of either compound introduce a long‐term confounding impact on electrophysiological recordings [41, 53].

Given that alfaxalone prolongs GABA_A_‐R‐mediated inhibitory currents, the anesthetic represents a potential confounding factor for electrophysiological recordings. Considering that the GABA_A_‐R plays an integral role in the synaptic arrest model of anoxia tolerance in the common goldfish, determining a sufficiently short washout period is essential to support its use in brain anoxia tolerance research.

The goal of our study is to determine if alfaxalone sedation will have a sufficiently short washout period from telencephalic brain slices to make same‐day electrophysiological investigations in goldfish brain practical. Since it is a GABA_A_‐R agonist it should have no long‐term impact on passive and active action potential or GABA_A_‐R current electrophysiological properties. Furthermore, alfaxalone sedation prior to decapitation will substantially improve animal welfare when anoxia‐tolerant species are used for electrophysiological experiments.

## Materials and methods

### Animal care and cortical sheet preparation

This study was approved by the University of Toronto Animal Care Committee and conforms to the care and handling of animals as outlined in the Canadian Council on Animal Care's Guide to the Care and Use of Experimental Animals, Vol. 2. Animal use protocol number (20012745). Common goldfish, *Carassius auratus*, weighing 50–150 g, were held in flowing dechlorinated City of Toronto tap water at 18 °C at the University of Toronto. Whole‐animal alfaxalone exposure (alfaxalone sedated goldfish) was achieved through immersing the goldfish in 500 mL of water containing 0.5 mL of 10 mg·mL^−1^ alfaxalone, resulting in a final concentration of 0.01 mg·mL^−1^ (Alfaxan® Multidose, Jurox Pty. Ltd, Rutherford, NSW, Australia). Once deep anesthesia was achieved, as assessed through the loss of the righting reflex, the animal was decapitated, and the whole brain was removed from the cranium and placed in alfaxalone‐free oxygenated ice‐cold 4 °C artificial cerebrospinal fluid (aCSF) containing (in mmol·L^−1^): 20 NaHCO_3_,118 NaCl, 1.2 MgCl_2_·6H_2_O, 1.2 KH_2_PO_4_, 1.9 KCl, 10 HEPES‐Na, 10 D‐glucose and 2.4 CaCl_2_ (pH 7.6, adjusted with HCl); osmolarity 285–290 mOsm. Both lobes of telencephalon were then dissected from the rest of the brain in the chilled aCSF. Naïve goldfish tissue was defined as tissue harvested from non‐alfaxalone exposed goldfish and alfaxalone sedated goldfish tissue was used to define tissue harvested from whole‐animal alfaxalone sedated goldfish as described above. Decapitation and whole brain removal remained the same for both groups. Each telencephalon lobe was glued to a sectioning plate on a vibratome slicing chamber using cyanoacrylate glue (Krazy Glue) and was filled with alfaxalone‐free 4 °C aCSF. Isolated goldfish telencephalon was cut into 400–300 μm slices using a VT1200S vibratome. Slices were lifted out of the chamber and were stored in vials of alfaxalone‐free aCSF for no longer than 48 h [54]. Slice washing began following slice preparation.

### Whole‐cell electrophysiology techniques

Goldfish telencephalon slices were perfused with oxygenated aCSF (99% O_2_, 1% CO_2_), and whole‐cell recordings were performed using the voltage‐clamp method with 8–12 MΩ borosilicate glass pipette electrodes (Harvard Apparatus Ltd, Holliston, MA, USA) containing the following (in mmol·L^−1^): 120 potassium gluconate, 10 KCl, 15 sucrose, 2 Na_2_ATP, 0.44 CaCl_2_, 0.75 EGTA and 10 HEPES‐Na (pH 7.6 adjusted with KOH); osmolarity 285–290 mOsm.

For goldfish slices, cell‐attached 1–20 GΩ seals were obtained using the blind‐patch technique [55]. To achieve a GΩ seal, the recording electrode was advanced toward the cell using a PCS‐6000 motorized manipulator (Burleigh, Newton, NJ, USA) until the square‐wave pulse was abruptly decreased, at which point a slight negative pressure was applied to form a seal. To break into the cell, a soft pulse of negative pressure was applied to break through the cell membrane, while potential was voltage‐clamped to −55 mV. Once the whole‐cell configuration was established, cells were given at least 2 min to acclimate to experimental conditions before access resistance was measured, which normally ranged from 20 to 30 MΩ. Patches were discarded if access resistance varied by > 25% over the course of an experiment. Data was collected at 5–10 kHz using a MultiClamp 700B digital amplifier, a CV‐7B head stage, and a Digidata 1550B digitizer (Molecular Devices, Sunnyvale, CA, USA) and then collected and stored on computer using clampex 11.2 software (Molecular Devices). A liquid junction potential (LJP) was accounted for and is experimentally measured between the aCSF and the pipette solution and supported by LJP calculations using a generalized version of the Henderson equation (Clampex junction potential calculator; Molecular Device) [37, 56].

### Electrophysiological identification and measurement of action potential parameters

**Fig. 1:**
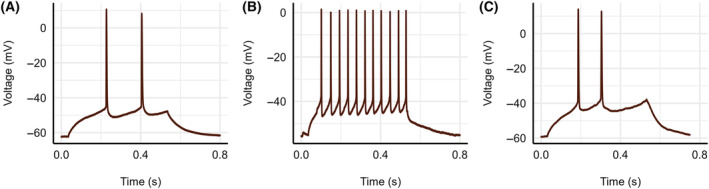
Induced action potential recordings. AP traces generated from 500 ms current steps in 10 pA increments from a naïve tissue (A) pyramidal neuron and (B) stellate neuron, and (C) a pyramidal neuron from goldfish tissue following whole‐animal alfaxalone sedation.

Pyramidal neurons were studied and characterized based on electrophysiological properties. In current clamp mode when current was injected, the more common pyramidal neurons exhibited spike frequency adaptation in response to sustained current, this is not seen in stellate neurons (Fig. 1A). The less abundant, rapidly firing stellate cell action potentials were demonstrated to have smooth rising and falling sections followed by a voltage undershoot, as depicted by Fig. 1B [57].

Action potential threshold (APth) was determined by current‐clamping cells and injecting currents in 10 pA increments in a stepwise manner from sub‐threshold for 500 ms until a spike was elicited. Threshold was recorded at the point at which a sharp elevation in voltage was observed. The full spike amplitude was measured from the point of the APth to the spike tip, while the half‐amplitude spike width was measured as the time elapsed between the two‐points of the half‐amplitude on the spike. The rising time of a spike was calculated as the time elapsed between 10% of the full spike amplitude to 90% of the full spike amplitude, while the decay time was calculated as 90% of the full spike amplitude to 10% of the full spike amplitude. Whole‐cell conductance was measured by voltage‐clamping cells and generating a voltage‐ramp, through increasing voltage from −120 to −50 mV for 150 ms. Data measurements for all parameters were made with clampfit software (Axon Instrument) [56].

### Impact of acute alfaxalone application on evoked naive tissue GABA_A_‐R current

To initiate a GABA_A_‐R current, the same methodology for whole‐cell recordings was used with naïve goldfish telencephalon slices, neurons were voltage clamped at a holding potential of −80 mV, and 2 mm GABA was applied for 1–2 s [54]. These changes resulted in large outward GABA_A_‐R currents that were easily detected and differentiated from other currents. From the GABA_A_‐R currents produced, decay time was measured as the time elapsed between 90% and 10% of the peak amplitude, area under the curve was measured as the integrated area between the measured current and baseline, the peak amplitude was measured, and the baseline holding current was measured, using clampfit software (Axon Instrument). Electrophysiological properties were normalized to whole‐cell capacitance. Tissue slices were then perfused with oxygenated aCSF and 1 μm alfaxalone for 15 min where the same protocol was repeated to measure GABA_A_‐R current decay time, integrated area under the curve, peak amplitude, and baseline holding current. 1 μm alfaxalone was produced through adding 2 μL of stock alfaxalone solution into 60 mL of control aCSF. This procedure was repeated utilizing oxygenated aCSF without added alfaxalone (control solution) as a negative control and 100 μm picrotoxin, a general GABA_A_‐R antagonist, as a positive control to elicit a shift in baseline holding current [58, 59]. Alfaxalone‐free stock solution (vehicle, a generous gift from Jurox) had no significant impact when applied alone (data not shown) (*n* = 4).

### GABA_A_‐R reversal potential determination

To determine the E_GABA_, cells were perfused with 1 μm of the voltage‐gated sodium channel inhibitor, tetrodotoxin (TTX), for approximately 5 min to eliminate the generation of action potentials. Current–voltage (I/V) curves were then constructed through voltage‐clamping naïve goldfish tissue pyramidal neurons and generating a voltage‐ramp, through increasing voltage from −90 to −30 mV for 150 ms and measuring the corresponding current under control/baseline conditions and approximately 2–3 s following GABA perfusion.

### Impact of whole‐animal alfaxalone sedation on evoked GABA_A_‐R current

Large outward GABA_A_‐R currents were initiated using the same methodology as stated prior. GABA_A_‐R current decay time, integrated area under the curve, and peak amplitude were determined using the same methodology as stated prior. Decay time, integrated area under the curve, peak amplitude, and baseline holding current were then normalized to whole‐cell capacitance. The protocol was repeated with telencephalon slices obtained from alfaxalone sedated goldfish at 2, 3, and 4–6 h following alfaxalone application. The timepoints were binned so that any measurement performed between 1.5 and 2.5 h was considered 2 h following whole‐animal alfaxalone sedation, any measurement performed between 2.5 and 3.5 h was considered 3 h following whole‐animal alfaxalone sedation, and any measurement performed between 3.5 and 6.5 h was considered 4–6 h following whole‐animal alfaxalone sedation. Measurements made on the 30‐min mark were placed in the later time group. Following slice preparation, occurring 1.5 h after alfaxalone sedation, slices were washed every 30 min, with the aCSF housing the tissue slices being replaced with fresh alfaxalone‐free aCSF.

### Statistics

Welch's *t*‐tests were conducted to compare AP electrophysiological properties between treatments. AP traces which did not immediately return to baseline or hyperpolarize were excluded as decay time could not be determined accurately. Paired *t*‐tests were conducted to compare 0‐min GABA_A_‐R active and passive electrophysiological property values to 15‐min perfusion values, within treatment groups. Welch's *t*‐tests were conducted to compare absolute property values and the relative change in GABA_A_‐R active electrophysiological property values following 15‐min perfusion between treatment groups. A one‐way ANOVA was used to compare the absolute holding current values and relative changes in holding current values following 15‐min perfusion between treatment groups, followed by pairwise Welch's *t*‐tests. Welch's *t*‐tests were conducted to compare GABA_A_‐R electrophysiological property values at various timepoints following whole‐animal alfaxalone sedation, to naïve/control values. *N* values represent a recording from a single cell from a single telencephalic slide, from a single goldfish. *P* < 0.05 was considered statistically significant. All statistical analyses were performed using R.

## Results

### Action potential generation and structure analysis

To generate APs, 500 ms current steps in 10 pA increments were injected into cells, and voltage traces were obtained until APs were generated, at which point current injection was halted. Voltage traces containing one or multiple APs were then isolated for analysis. Current clamp recordings were conducted in naive/control tissue and tissue derived following alfaxalone sedation, as shown in Fig. 1A,C respectively, where pyramidal cell voltage traces were characterized by low frequency APs. AP traces lacking accommodation were identified as stellate neurons (Fig. 1B) and were not included in the present analysis. Passive voltage traces were generated to monitor neuronal membrane potential changes over time.

### Electrophysiological property analysis

**Fig. 2:**
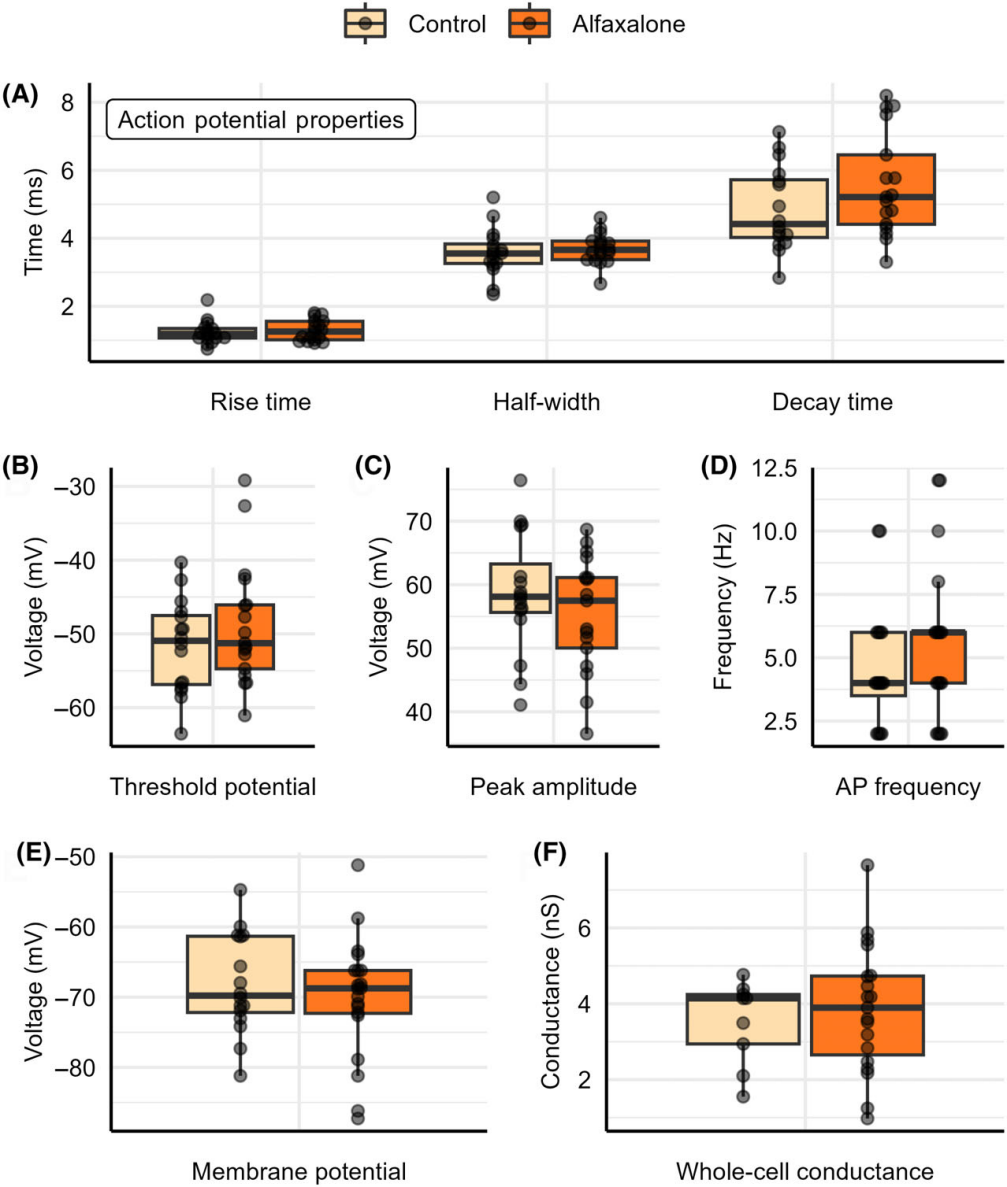
Electrophysiological properties for naive/control brain tissue and tissue derived following whole‐animal alfaxalone sedation. Properties include (A) rise time (ms), half‐width (ms), and decay time (ms) (*n* = 16 and 17) (B) threshold potential (mV) (*n* = 16 and 17) (C) peak amplitude (mV) (*n* = 16 and 17) (D) action potential frequency (Hz) (*n* = 16 and 17) (E) membrane potential (mV) (*n* = 16 and 20) (F) whole‐cell conductance (*n* = 9 and 19). Each point represents data from a separate experiment and *n* values are reported in terms of control and alfaxalone treated tissue respectively. Statistical significance was determined using Welch's *t*‐test.

To obtain AP electrophysiological characteristics from naïve/control tissue and tissue derived following whole‐animal alfaxalone sedation, data were analyzed using the program Clampfit. Peak amplitude, half‐width, rise time, decay time, membrane potential, action potential frequency and threshold potential were determined as summarized in Table 1. No statistical differences were found between control tissue and tissue derived following alfaxalone sedation for any of the electrophysiological properties, as demonstrated through Fig. 2A–E.

**Table 1:** Control and alfaxalone treated tissue AP electrophysiological property summary values. Data are mean ± SEM (*n* = 9–20).

Property	Control	Alfaxalone	*P*‐value
Rise time (ms)	1.23 ± 0.0843	1.29 ± 0.0749	0.495
Half‐width (ms)	3.58 ± 0.178	3.68 ± 0.108	0.271
Decay time (ms)	4.86 ± 0.309	5.58 ± 0.368	0.332
Threshold potential (mV)	−51.7 ± 1.59	−48.8 ± 2.05	0.614
Peak amplitude (mV)	58.6 ± 2.38	55.4 ± 2.23	0.643
Action potential frequency (Hz)	4.75 ± 0.629	5.65 ± 0.79	0.141
Membrane potential (mV)	−68.2 ± 1.77	−70.0 ± 1.91	0.382
Whole cell conductance (nS)	3.53 ± 0.370	3.86 ± 0.386	0.549

To determine whole‐cell conductance values for the obtained alfaxalone and control AP traces, voltage clamp recordings were conducted through a voltage ramp protocol, increasing voltage from −120 mV to −50 mV, while measuring corresponding current. Voltage and current measurements were plotted against each other in a I/V curve, linear regression of the I/V curves was used to generate a line of best fit, and the slope of the linear regression line was used to determine whole‐cell conductance values as summarized in Table 1. Demonstrated in Fig. 2F, no statistical differences were found between control and alfaxalone sedated goldfish tissue whole‐cell conductance values.

### GABA_A_‐R current generation and characterization

**Fig. 3:**
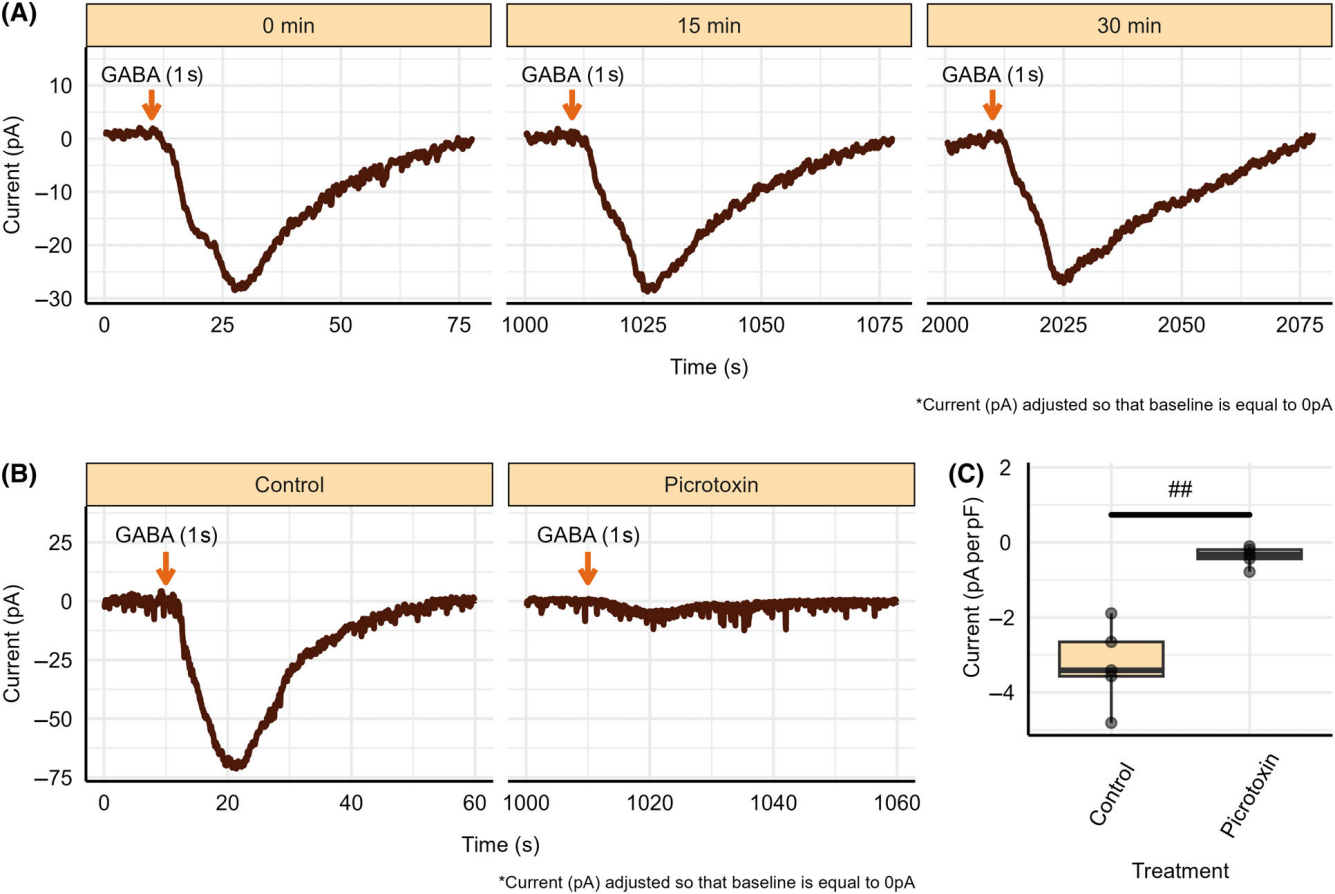
Whole‐cell GABA_A_‐R current characterization and stability. (A) Whole‐cell GABA_A_‐R currents taken 15 min apart. (B) GABA_A_‐R current trace following GABA application in control conditions, following 15 min of picrotoxin perfusion, and after 15 of aCSF perfusion recovery. (C) GABA_A_‐R current peak amplitude following GABA perfusion under control conditions and following 15 min of picrotoxin perfusion. Picrotoxin perfusion began immediately following the completion of the initial control GABA_A_‐R current. Each point represents data from a separate experiment (*n* = 5). Paired *t*‐test indicated significant difference from baseline/control conditions (^##^ *P* < 0.01).

To generate a whole‐cell GABA_A_‐R current trace, a GABA_A_‐R current was elicited through clamping the cell voltage at −80 mV and perfusing 2 mm GABA onto the tissue slice for 1–2 s. This process was repeated after 15 min, and 30 min using the same whole‐cell patch to generate additional GABA_A_‐R currents and confirm the long‐term stability of the whole‐cell patch, as shown in Fig. 3A. Whole‐cell patch recordings remained stable up to 30 min, as indicated by Fig. S1. Whole‐cell patch stability deceased beyond 30 min, however few recordings remained stable up to an hour. To confirm this was a GABA_A_‐R current, the antagonist picrotoxin was perfused into the recording chamber 15 min before GABA was applied. Following perfusion of aCSF containing 100 μm picrotoxin for 15 min, large GABA_A_‐R currents could not be generated (Fig. 3B), resulting in a significantly reduced GABA_A_‐R current peak amplitude normalized to whole‐cell capacitance (−3.27 ± 0.489 pA per pF vs. −0.368 ± 0.118 pA per pF, *P* = 0.00501, *n* = 5) (Fig. 3C).

### GABA_A_ reversal potential confirmation

**Fig. 4:**
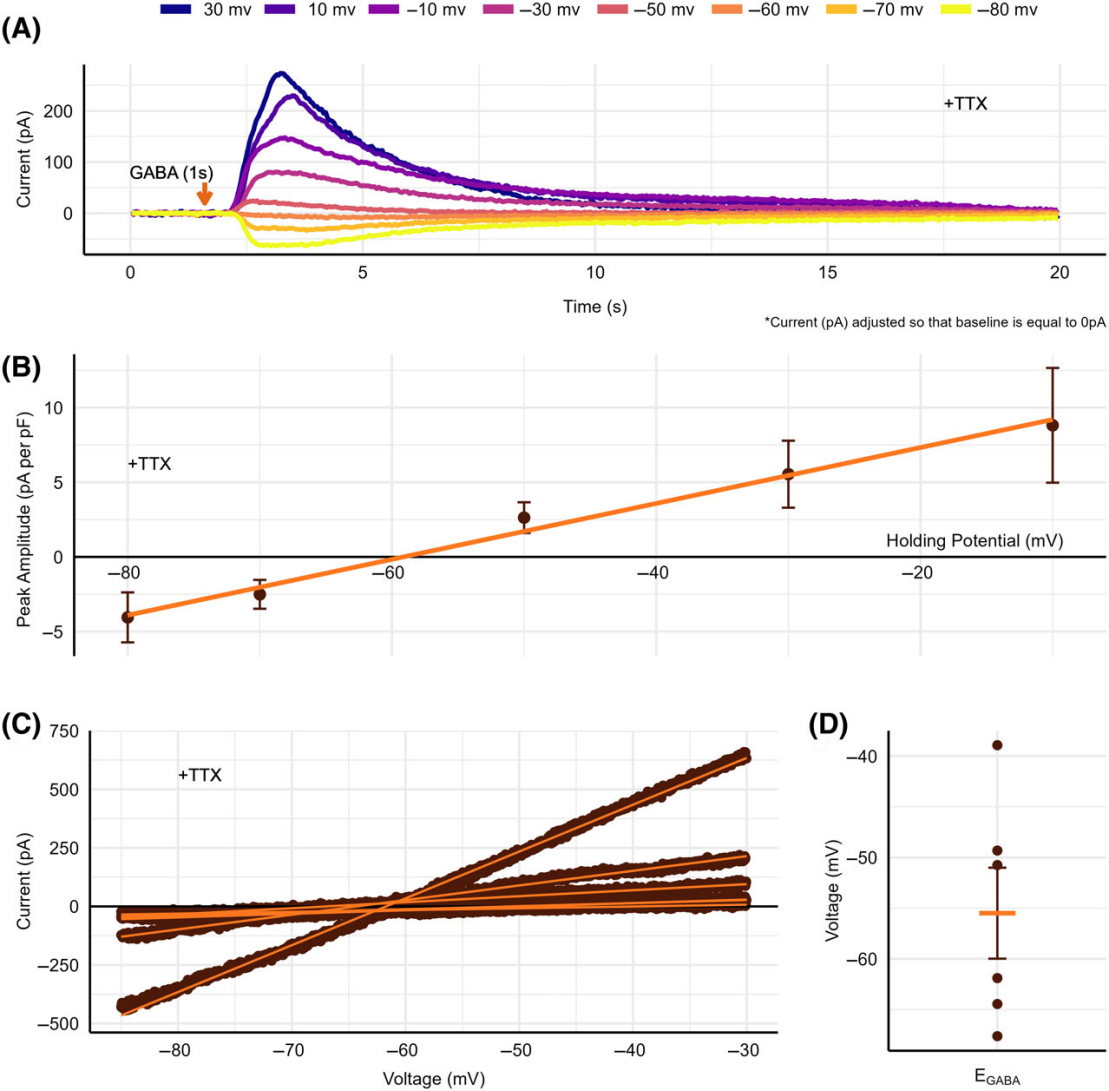
GABA_A_‐R reversal potential identification through generation of whole‐cell GABA_A_‐R mediated currents. (A) GABA_A_‐R currents at various holding potentials between −80 mV and 30 mV, demonstrating reverse directionality at potentials above the GABA_A_ channel reversal potential. (B) Whole‐cell current–voltage relationship depicting GABA_A_‐R current peak amplitude relative to holding potential. Peak amplitude increased linearly with holding potential. The x‐intercept represents E_GABA_. Values are means ± SEM (*n* = 5). (C) GABA‐specific whole‐cell current–voltage relationships taken in naïve goldfish tissue following exposure to 2 mm GABA (*n* = 6). (D) Average E_GABA_ value calculated through the x‐intercepts of GABA‐specific whole‐cell current–voltage relationships. E_GABA_ was calculated as mean ± SEM (*n* = 6).

To confirm the E_GABA_ value, a similar voltage clamp protocol was utilized to clamp the cell at various voltages between −80 mV to 30 mV in the presence of TTX, while measuring cell current. Whole‐cell GABA_A_‐R currents were elicited through perfusion of 2 mm GABA for 1–2 s. GABA_A_‐R currents with reversed directionality were recorded at potentials depolarized relative to E_GABA_ as demonstrated by Fig. 4A. Peak amplitude increased approximately linearly relative to holding potential as indicated by Fig. 4B.

To further confirm that the obtained current traces resulted from GABA_A_ channel activity, I/V curves were generated using a voltage ramp protocol in naïve goldfish tissue. These recordings were taken passively at baseline and following the steady perfusion of 2 mm GABA for 1–2 s, at the peak amplitude of the GABA_A_‐R whole‐cell current. The baseline current values were then subtracted from the peak amplitude values to obtain a GABA‐specific I/V curve, as demonstrated by Fig. 4C. The intersection between GABA‐specific I/V curve and the *x*‐axis represented the E_GABA_. E_GABA_ was calculated to be −55.49 ± 4.485 mV (*n* = 6), as indicated by Fig. 4D.

### Analysis of acute alfaxalone application onto naïve tissue

**Fig. 5:**
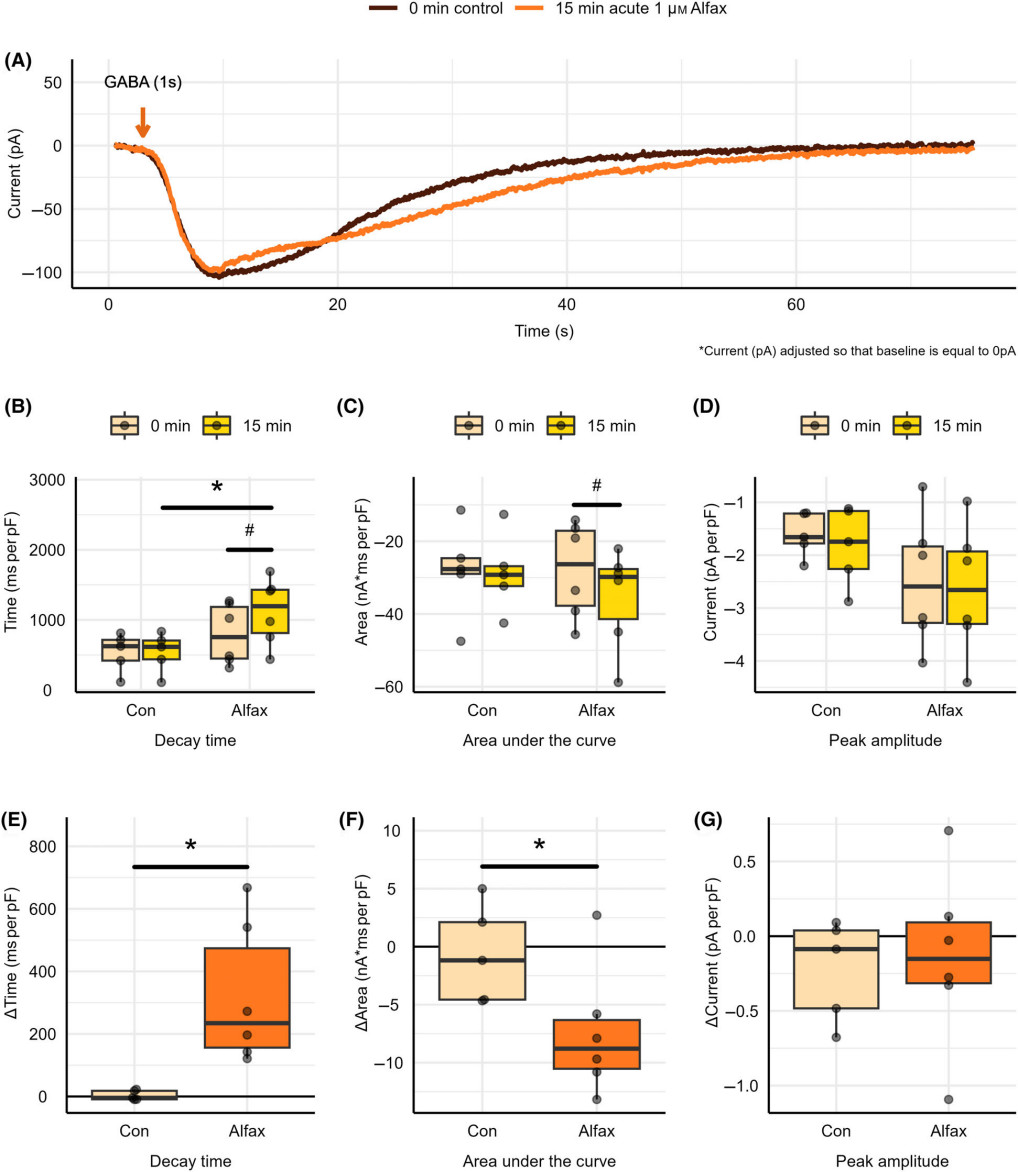
GABA_A_‐R potentiation following acute application of alfaxalone onto naive tissue. (A) GABA_A_‐R currents following GABA application at 0 min under control aCSF conditions and following 15 min of acute alfaxalone perfusion. Absolute (B) decay time, (C) area under the curve, and (D) peak amplitude at 0 min under control aCSF conditions and following 15 min of constant control aCSF (Con) or 1 μm alfaxalone (Alfax) perfusion onto naïve tissue. Change in (E) decay time, (F) area under the curve, and (G) peak amplitude at baseline and following 15 min of constant control aCSF or 1 μm alfaxalone perfusion onto naïve tissue. Each point represents data from a separate experiment (*n* = 5–6). Note negative y axes in (C), (D), (F), and (G) indicate increasing currents. Paired *t*‐tests indicated a significant difference from 0‐min recordings (^#^ *P* < 0.05). Welch's *t*‐tests indicated a significant difference in absolute property values or the change in property values between 15‐min control aCSF and acute alfaxalone recordings (*P < 0.05).

To confirm patch stability and determine the impact of alfaxalone on GABA_A_‐R currents, GABA_A_‐R currents were elicited in naïve tissue as described above, as shown in Fig. 5A. While maintaining the same whole‐cell patch, the cell was then perfused with either control aCSF or aCSF containing 1 μm alfaxalone for 15 min, and the same protocol as mentioned prior was utilized to generate an additional GABA_A_‐R trace as shown in Fig. 5A. An alfaxalone concentration of 1 μm was utilized based on the results of our dose–response curve analysis, as indicated by Fig. S2. Current decay time, area under the curve, and peak amplitude at 0 min and following 15 min of control aCSF perfusion or the acute application of alfaxalone were determined using the program Clampfit and were normalized to whole‐cell capacitance.

A statistically significant increase in decay time was found following 15 min of acute alfaxalone application, in comparison to 0‐min recordings, as indicated by Fig. 5B (795 ± 176 ms per pF to 1120 ± 194 ms per pF, *P* = 0.0175, *n* = 6), representing a 51.8 ± 16.9% change. A statistically significant difference in area under the curve was found following 15 min of acute alfaxalone application, in comparison to 0‐min recordings, as indicated by Fig. 5C (−28 ± 5.38 nA*ms per pF to −35.4 ± 5.62 nA*ms per pF, *P* = 0.0221, *n* = 6), representing a 34.6 ± 11.4% change. No statistically significant (−2.5 ± 0.50 pA per pF to −2.65 ± 0.502 pA per pF, *P* = 0.569 *n* = 6) difference in peak amplitude was found following 15 min of acute alfaxalone application, in comparison to 0‐min recordings as shown in Fig. 5D.

A statistically significant increase in absolute decay time values was found between 15 min of control aCSF perfusion and 15 min of acute alfaxalone perfusion, as shown in Fig. 5B (540 ± 126 ms per pF vs. 1120 ± 194 ms per pF, *P* = 0.0360 *n* = 5–6). No statistically significant difference in the absolute area under the curve (−28.7 ± 4.83 nA*ms per pF vs. −35.4 ± 5.62 nA*ms per pF, *P* = 0.386 *n* = 5–6) or peak amplitude (−1.83 ± 0.334 pA per pF vs. −2.65 ± 0.502 pA per pF, *P* = 0.211 *n* = 5–6) values was found between 15 min of control aCSF perfusion and 15 min of acute alfaxalone perfusion, as shown in Fig. 5C,D respectively. A statistically significant difference in the change in decay time was found between 15 min of control aCSF perfusion and 15 min of acute alfaxalone perfusion, as shown in Fig. 5E (3.58 ± 6.98 ms per pF vs. 324 ± 92.8 ms per pF, *P* = 0.0181 *n* = 5–6). Further, a statistically significant difference in the change in area under the curve was found between 15 min of control aCSF perfusion and 15 min of acute alfaxalone perfusion, as shown in Fig. 5F (−0.661 ± 1.89 nA*ms per pF vs. −7.44 ± 2.27 nA*ms per pF, *P* = 0.0475 *n* = 5–6). No statistically significant difference in the change in peak amplitude following 15 min of perfusion was found between control and acute alfaxalone conditions, as shown in Fig. 5G (−0.223 ± 0.152 pA per pF vs. −0.148 ± 0.243 pA per pF, *P* = 0.853 *n* = 5–6).

**Fig. 6:**
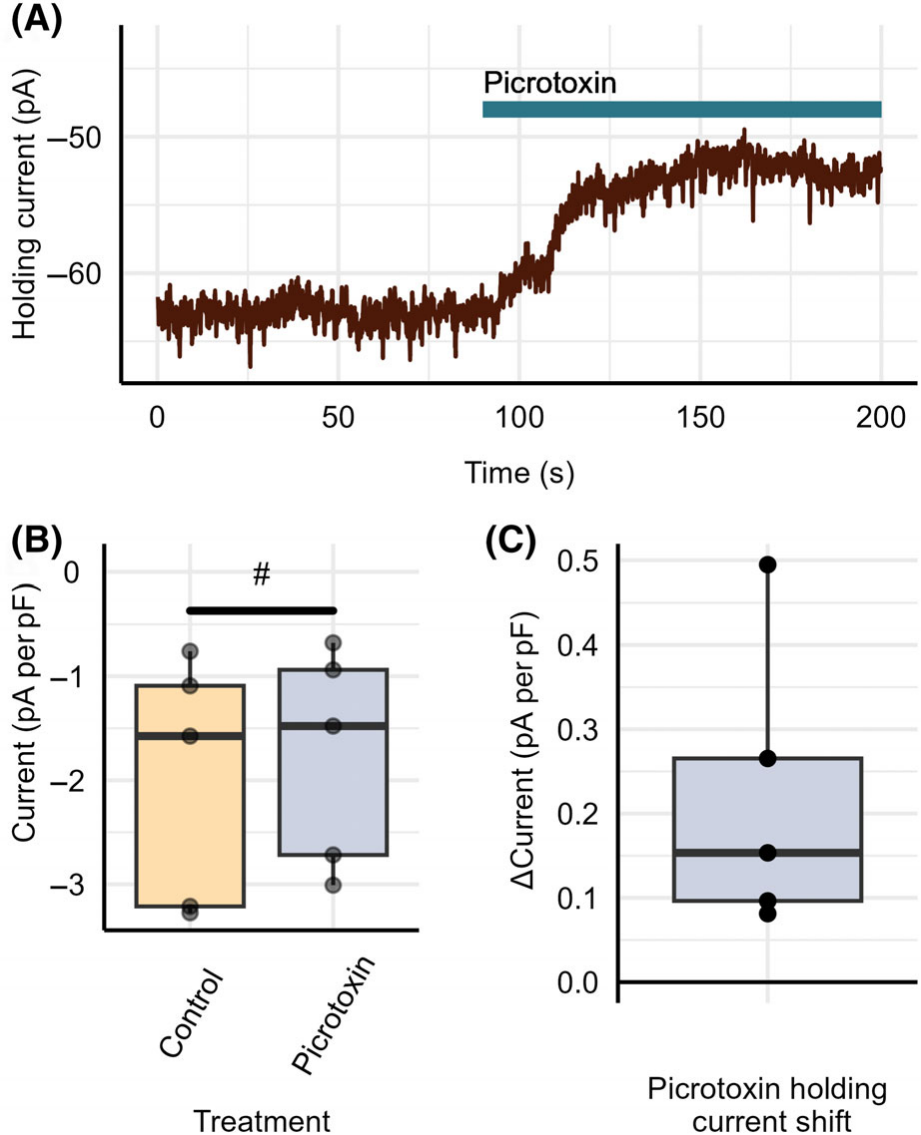
Picrotoxin induces a positive shift in holding current following application onto naïve tissue. (A) Positive shift in holding current following the application of general GABA_A_‐R antagonist picrotoxin at a holding potential of −80 mV. Absolute change (B) and difference (C) in holding current following the application of 1 μm alfaxalone and 100 μm picrotoxin. Each point represents data from a separate experiment (*n* = 5). Paired *t*‐test indicated significant difference from baseline holding current (^#^ *P* < 0.05).

To confirm the presence of tonic GABA_A_‐R activity, holding current required to maintain a cellular membrane potential of −80 mV was measured under control/baseline conditions and following the application of the general GABA_A_‐R antagonist, picrotoxin, in naïve tissue as shown in Fig. 6A. Following the application of 100 μm picrotoxin, a statistically significant shift in holding current (0.218 pA per pF ± 0.0763pA per pF, *n* = 5, *P* = 0.0460) was found, as shown in Fig. 6B,C.

**Fig. 7:**
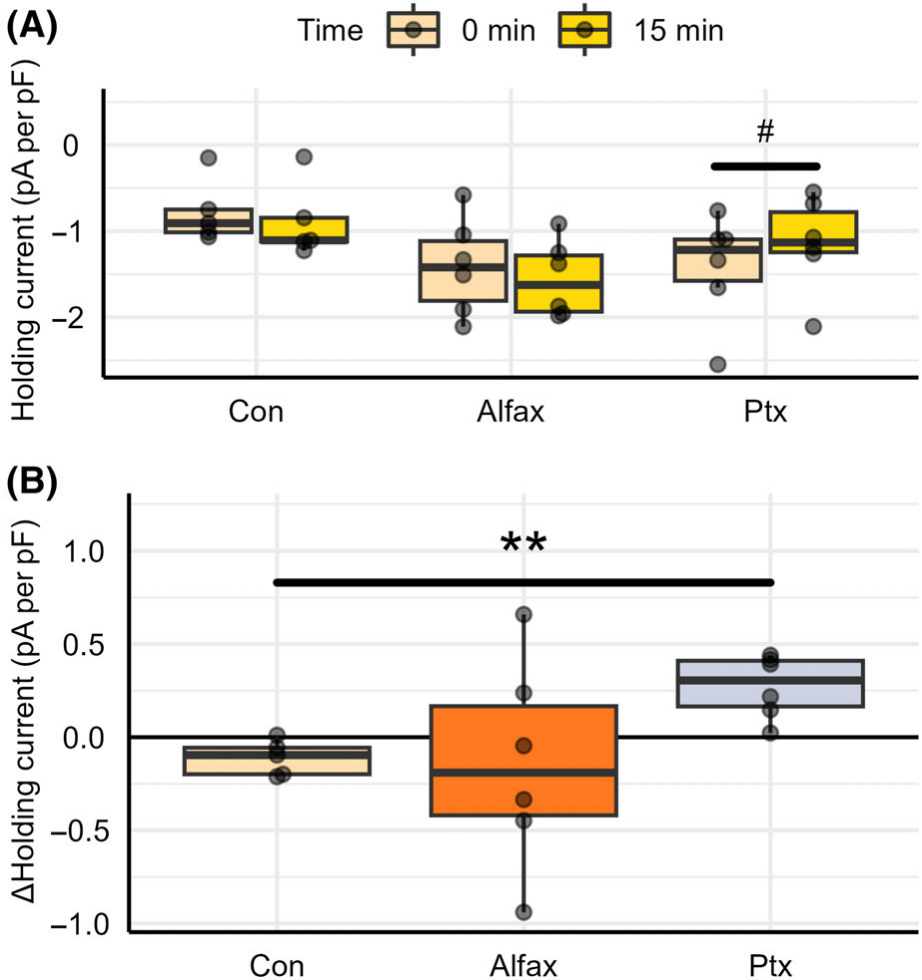
Alfaxalone induces no significant change in holding current following application onto naïve tissue. (A) Absolute and (B) change in holding current following the application of control aCSF (Con), 1 μm alfaxalone (Alfax), and 100 μm picrotoxin (Ptx). Each point represents data from a separate experiment (*n* = 5–6). Paired *t*‐tests indicated a significant difference from 0‐min recordings (^#^ *P* < 0.05). Pairwise Welch's *t*‐tests indicated a significant difference in absolute holding current or change in holding current between 15‐min recordings (**P < 0.01).

To determine any passive impact of alfaxalone on the holding current required to maintain a cellular membrane potential of −80 mV, the holding current was measured under control conditions and following acute application of 1 μm alfaxalone onto naïve tissue for 15 min. As a positive control, holding current was measured under control conditions and following the acute application of 100 μm picrotoxin. Holding current was determined using the program Clampfit and was normalized to whole‐cell capacitance. No statistically significant difference in holding potential was found between 0‐ and 15‐min recordings under normal aCSF control conditions (−0.778 ± 0.166 pA per pF to −0.888 ± 0.198 pA per pF, *P* = 0.0599, *n* = 5) and following the acute application of alfaxalone (−1.41 ± 0.228 pA per pF to −1.56 ± 0.182 pA per pF, *P* = 0.530, *n* = 6), as indicated by Fig. 7A. A statistically significant shift in holding current (−1.42 ± 0.256 pA per pF to −1.14 ± 0.225 pA per pF, *P* = 0.0110, *n* = 6) was found between control and 100 μm picrotoxin application treatment groups as indicated by Fig. 7A.

Using a one‐way ANOVA, no statistically significant difference in the absolute holding current values were found at 15 min between the three conditions (*P* = 0.0985, *n* = 5–6). Using a one‐way ANOVA, a statistically significant difference in the change in holding current was found at 15 min between the three conditions (*P* = 0. 0.00530, *n* = 5–6). Following a pairwise analysis, a statistically significant increase in the change holding current was found between control aCSF conditions and picrotoxin treatment (−0.11 ± 0.0424 pA per pF vs. 0.273 ± 0.0693 pA per pF, *P* = 0.00447, *n* = 5–6), as shown in Fig. 7B. Further, no statistically significant difference was found in the change in holding current between control aCSF conditions and alfaxalone treatment (−0.11 ± 0.0424, *P* = 0.886, *n* = 5–6), or between alfaxalone treatment and picrotoxin treatment (*P* = 0.262, *n* = 6), as shown in Fig. 7B.

### Alfaxalone washout time

**Fig. 8:**
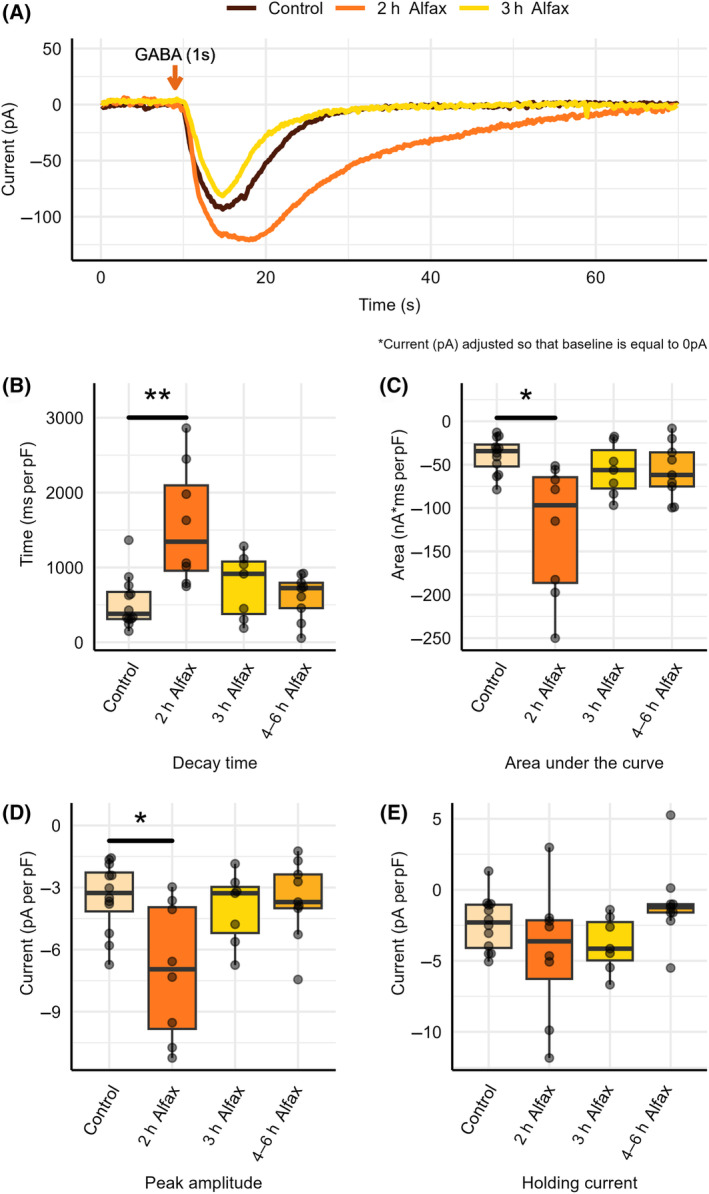
GABA_A_‐R potentiation induced through whole‐animal alfaxalone sedation reversed at 3 h following application. (A) GABA_A_‐R current following GABA application in control conditions, 2 h following alfaxalone application, and 3 h following alfaxalone application. GABA_A_‐R current (B) decay time (C) area under the curve (D) peak amplitude and (E) holding current in control conditions (*n* = 12) and at 2 h (*n* = 8), 3 h (*n* = 7), and 4–6 h (*n* = 9), following whole‐animal alfaxalone sedation. Each point represents data from a separate experiment. Welch's *t*‐test indicated significant difference from control conditions (*P < 0.05, **P < 0.01).

To determine the washout period of alfaxalone, GABA_A_‐R currents were measured as described above in naive/control tissue and tissue harvested from whole‐animal alfaxalone sedation at various time‐points following anesthetic administration, as shown in Fig. 8A. Current decay time, area under the curve, and peak amplitude were determined using the program Clampfit and were normalized to whole‐cell capacitance.

As shown in Fig. 8B, a statistically significant increase (*P* = 0.00757) in decay time was found between control (531 ± 99.3 ms per pF, *n* = 12) conditions and 2 h following alfaxalone application (1570 ± 283 ms per pF, *n* = 8). This statistically significant increase in decay time was reversed at 3 h (757 ± 164 ms per pF, *n* = 7, *P* = 0.264) and 4–6 h following alfaxalone sedation (606 ± 99.1 ms per pF, *n* = 9, *P* = 0.597).

As shown in Fig. 8C, a statistically significant increase (*P* = 0.0148) in area under the curve was found between control (−39.2 ± 5.92 nA*ms per pF, *n* = 12) conditions and 2 h following alfaxalone application (−125 ± 26.7 nA*ms per pF, *n* = 8). This statistically significant increase in area under the curve was reversed at 3 h (−56.0 ± 11.5 nA*ms per pF, *n* = 7, *P* = 0.225) and 4–6 h following alfaxalone sedation (−57.2 ± 10.9 nA*ms per pF, *n* = 9, *P* = 0.171).

As shown in Fig. 8D, a statistically significant increase (*P* = 0.0190) in peak amplitude was found between control (−3.47 ± 0.486 pA per pF, *n* = 12) conditions and 2 h following alfaxalone application (−7.01 ± 1.16 pA per pF, *n* = 8). This statistically significant increase in peak amplitude was reversed at 3 h (−4.03 ± 0.657 pA per pF, *n* = 7, *P* = 0.507) and 4–6 h following alfaxalone sedation (−3.59 ± 0.638 pA per pF, *n* = 9, *P* = 0.881).

To determine whether alfaxalone had any long‐term impact on GABA_A_‐R mediated tonic currents, holding current was measured in control tissue and alfaxalone‐treated tissue at various time‐points following anesthetic administration. Holding currents were determined using the program Clampfit and were normalized to whole‐cell capacitance. As shown in Fig. 8E, no statistically significant difference in holding current was found between control (−2.4 ± 0.546 pA per pF, *n* = 12) conditions and 2 h (−4.41 ± 1.66 pA per pF, *n* = 8, *P* = 0.282), 3 h (−3.82 ± 0.730 pA per pF, *n* = 7, *P* = 0.145), or 4–6 h (−0.973 ± 0.934 pA per pF, *n* = 9, *P* = 0.209) following alfaxalone sedation.

## Discussion

### Alfaxalone has no long‐term impact on action potential properties

In this study, the impact of whole‐animal alfaxalone sedation on action potential electrophysiological characteristics was investigated using whole‐cell patch recordings. It was shown that whole‐animal alfaxalone sedation has no significant impact on any of the evoked action potential properties analyzed in this study, including rise time, decay time, half‐width time, peak amplitude, threshold potential, alongside membrane potential and whole cell conductance. Previous average membrane potential and whole‐cell conductance values for the common goldfish were found to be −72.18 ± 2.3 mV and 3.96 ± 0.95 nS respectively [54]. Similar average membrane potential and whole‐cell conductance values were later found to be −72.33 ± 3.35 mV and 3.95 ± 0.36 nS respectively [60]. In comparison, our study found average membrane potential and whole‐cell conductance values of −68.2 ± 1.77 mV and 3.53 ± 0.370 nS in naïve/control tissue and − 70.0 ± 1.91 mV and 3.86 ± 0.386 nS in tissue derived following whole‐animal alfaxalone sedation.

Similar rise time, decay time, and half‐width time in both alfaxalone sedated and control goldfish tissue was expected, considering the mechanism in which the anesthetic induces sedation. While alfaxalone is defined as a GABA_A_ agonist, the electrophysiological properties, rise time, decay time, and half‐width time, are determined by the action of voltage‐sensitive fast sodium currents and outward potassium currents [61]. Alfaxalone has not been found to alter Na^+^ and K^+^ channel structure or conductance following its application, and therefore our results support the proposed mechanism regarding alfaxalone binding and action [3].

### Prolonged GABA_A_ ‐mediated currents and their antagonistic elimination

In order to analyze GABA_A_‐R current electrophysiological properties, it was important to first confirm the long‐term stability of whole‐cell patch‐clamp recordings following the perfusion of GABA multiple times over an extended period of time. The stability of whole‐cell patch recordings up to 30 min was confirmed following the generation of two GABA_A_‐R currents, at two timepoints separated by 15 min. These large GABA_A_‐R currents were the result of extrasynaptic GABA_A_‐R activity, as indicated by the length of the measured decay times. The average decay time of synaptic inhibitory postsynaptic currents in rat hippocampal neurons was found to be 41 ± 9 ms [7], in comparison to the GABA_A_‐R currents produced in this study, which demonstrated a much larger average decay time of 531 ± 99.3 ms per pF in naïve tissue. Stability was dependent on a holding potential of −80 mV, and the use of physiologically relevant concentrations of Cl^−^, rather than a high intracellular‐chloride solution to mediate large outward GABA_A_‐R currents. The large negative cell holding potential allowed for visible outward chloride currents, while preventing the opening of voltage‐dependent Na^+^ and NMDA receptor channels [62]. The resulting phasic GABA_A_‐R currents followed the expected biphasic decay response caused by the fast and slow deactivation phases [63, 64]. Desensitization at the single‐channel level is described as the process where the GABA_A_ channel is unable to open despite the presence of GABA. In this desensitized state, the GABA_A_‐R can retain GABA, and if GABA remains bound to the receptor following this refractory period, the channel is able to immediately open once again [64, 65, 66]. The fast components of GABA_A_‐R desensitization correspond to receptors displaying a rapid refractory period, allowing these components to remain bound to GABA and to immediately reopen following the completion of this refractory period [67]. As such, the fast components of GABA_A_‐R desensitization can prolong whole‐cell GABA_A_‐R currents, causing the slow phase of deactivation [64, 66].

Additionally, few recordings depicted a GABA_A_‐R current including a deactivation period which eventually reached a plateau, hyperpolarized relative to the baseline. The recorded current measurement would then approach baseline over a much longer period of time. Decay time was defined as the time between 90% and 10% of the peak amplitude to account for this occurrence; however, some recordings included a plateau that significantly deviated from the baseline. While these recordings were not included in the overall comparison, their occurrence can be explained through two possible mechanisms. GABA spillover into extracellular space causes the delayed activation of perisynaptic GABA_A_‐Rs, thus resulting in a prolonged phasic GABA_A_‐R response [64, 68]. Additionally, to accompany the phasic GABA response, slowly activated, low‐amplitude, tonic receptors also play a role, especially in the extrasynaptic response to GABA. Explained by their limited desensitization, tonic receptors exhibit GABA responses that can last seconds to minutes, and also exhibit chronic activation [64, 69]. The presence of tonic GABA_A_‐Rs was first identified through the use of GABA_A_ antagonists bicuculline and gabazine, which both reduced the holding current required to maintain a desired membrane potential [70]. The activation of either extra‐ or perisynaptic GABA receptors may have contributed to the prolonged return to baseline through the spillover of GABA into the extracellular space, thus representing a limitation to our study.

Alfaxalone selectively binds to GABA_A_‐R channels, therefore it was also important to ensure that the GABA_A_‐R currents were the result of GABA_A_‐R activity, as opposed to one of the other three GABA‐R subtypes, including GABA_B_ and GABA_C_ [71]. Our study confirmed that the recorded GABA_A_‐R currents were evoked through GABA_A_‐mediated channel activity, using three separate methods of confirmation. First, by measuring whole cell conductance at baseline and immediately following the perfusion of GABA, the E_GABA_ was identified through interpolating the point at which the two traces intersected. In naïve goldfish tissue, an E_GABA_ value of −55.5 ± 4.49 mV was calculated, similar to the previously calculated value of −53.80 ± 2.63 mV, thus providing support that the currents were likely the result of GABA_A_‐R channel activity [54]. Second, this reversal potential was then confirmed, as current directionality reversed as expected after passing through E_GABA_ and producing a GABA_A_‐R current at holding potentials depolarized relative to E_GABA_. As a third and final method of confirmation, the negative control experiment was performed by eliminating the GABA_A_‐R‐mediated response to GABA application with the agonist picrotoxin. Perfusion of GABA_A_‐R antagonist, gabazine in turtle cortical sheets was previously found to completely eliminate the GABA_A_‐R‐mediated response to GABA perfusion in a similar manner [37].

Beyond the classification of the GABA‐R channel subtype, the GABA_A_‐R can be further broken town into individual subtypes, composed of different units. Within the human genome 19 GABA_A_ subunits have been identified, divided into classes (α, β, γ, ρ, θ, ε, π and δ) and their respective isoforms (α1–6, β1–3, γ1–3 and ρ1–3) [72]. Utilizing these 19 subunits, upwards of 500 subtypes of functional GABA_A_‐R channels are estimated to exist [64, 73]. Furthermore, cells can co‐express multiple GABA_A_‐R subunit isoforms, and therefore subtypes, dependent on cellular location [74]. The future identification and analysis of prominent GABA_A_ subtypes which are expressed in the common goldfish pyramidal neurons at the single channel level would allow for a further understanding of the composition of whole‐cell GABA_A_‐R currents. Furthermore, a better understanding of prominent subunits expressed within goldfish pyramidal neurons would help explain the whole‐cell response to alfaxalone, as the anesthetic produces variable allosteric modification dependent effects on the GABA_A_ subunits present. Alfaxalone induces the most effective modulation on the α1β1γ2L GABA_A_‐R isoform [75].

### Alfaxalone has a short‐term impact on GABA_A_‐R current properties

The short‐term alfaxalone‐induced GABA_A_‐R current potentiation was explored through acutely perfusing alfaxalone onto naïve tissue and comparing values at 0 min, or baseline values, to 15 min of anesthetic application. Alfaxalone had a significant impact on increasing GABA_A_‐R current decay times and area under the curve relative to 0‐min values, however no significant change in peak amplitude was found. These results were consistent when comparing the change in decay time and area under the curve at 15 min following acute alfaxalone application in comparison to 15 min of control aCSF perfusion. However, when considering absolute property values at 15 min of acute alfaxalone or control aCSF perfusion, only a significant increase in decay time was found. In addition, alfaxalone‐free stock aCSF solution induced no significant impact. Consistent with our results, the alfaxalone solvent, 2‐hydroxypropyl‐β‐cyclodextrin, has been shown to induce no significant impact when applied alone, following dilution by a factor of 1000 [76]. Stock alfaxalone was diluted by a factor of at least 20 000 in our study. Overall, our findings confirm that alfaxalone induces short‐term potentiation of the GABA_A_‐R channel, supporting previous studies which identified that the same concentration of acute alfaxalone treatment (1 μm) prolonged the mean open‐time of GABA_A_‐R channels [6, 77]. Studies in cultured rat hippocampal neurons have shown a similar increase in decay time for spontaneous and induced GABA‐mediated inhibitory postsynaptic currents [7, 78]. Furthermore, the study by Harrison *et al*. [78] presented similar results when comparing decay time, peak amplitude, and area under the curve before and during alfaxalone application. Paralleling our findings, an increase in inhibitory post synaptic current decay time and charge transfer (area under the curve) was found during acute alfaxalone application and peak amplitude either remained the same or slightly decreased.

Additionally, no significant change in holding current was seen following the perfusion of 1 μm alfaxalone for 15 min onto naïve tissue, indicating that alfaxalone did not potentiate tonic GABA_A_‐R currents. As alfaxalone induces differing modulation depending on the GABA_A_ subunits present [75] this result could be explained by the subunit composition of extracellular tonic GABA_A_ receptors, which differ from phasic GABA_A_ receptors [64, 79]. Alternatively, it could be explained by the long duration of tonic receptor modulation, which occurs over the span of seconds to minutes. The subtypes involved in tonic GABA_A_ receptors have a high affinity for GABA and incomplete desensitization, leading to steady, low‐level activation. The increased GABA_A_‐R open channel probability and stabilization of the open state induced by alfaxalone may have a limited effect on tonic receptors which maintain a steady open state [64, 80].

In contrast to alfaxalone application, a statistically significant increase in holding current was seen following the perfusion of 100 μm picrotoxin, a non‐competitive synaptic, peri‐synaptic and extra‐synaptic tonic GABA_A_‐R channel blocker [58]. This result is consistent with studies in the western painted turtle, which demonstrated a similar shift in holding current following the application of the general GABA_A_‐R channel blocker bicuculline methiodide [81]. Bicuculline application is known to induce a similar shift in holding current as picrotoxin [59]. Furthermore, the significant increase in holding current following picrotoxin perfusion supports the presence of extrasynaptic tonic GABA_A_‐R activity in the common goldfish pyramidal neurons.

Alfaxalone potentiated decay time and area under the curve but the effect saturated at concentrations of approximately 1 μm or greater, as indicated by Fig. S2. Additionally, peak amplitude demonstrated a poor fit to the Hill equation, with minimal potentiation at any alfaxalone concentration, which supports our finding that acute alfaxalone application has no significant impact on potentiating GABA_A_‐R current peak amplitude at concentrations between 0.1–1.5 μm. Successful whole‐cell patch clamp recordings at a 1 μm alfaxalone concentration falls within the range of concentrations (0.1–1 μm) used in previous experiments in cultured rat hippocampal neurons where a similar increase in GABA_A_‐R decay times and charge transfer (area under the curve) was found [78]. Analysis of electrophysiological properties following the acute application of alfaxalone onto naïve tissue was limited at higher anesthetic concentrations. Alfaxalone concentrations greater than 1.5 μm destabilized whole‐cell patch recordings beyond approximately 10 min of acute perfusion. Alfaxalone increases liposome membrane fluidity in a concentration‐dependent manner, with membrane fluidity showing a positive correlation with anesthetic concentration [82]. This disruption to membrane stability may explain patch destabilization at greater alfaxalone concentrations and could set the upper limits of the dose–response curve. Furthermore, previous studies analyzing lengthened inhibitory post synaptic current decay times in the presence of high concentrations (10 μm) of alfaxalone used paired recordings at much shorter intervals (1 min) than our study [78]. Stable whole cell patches following the application of acute alfaxalone treatment at concentrations greater than 1 μm were possible in our study at timepoints less than 10 min. However, in our study we used 15 min between GABA_A_‐R current recordings to ensure washout of GABA from the bath and for alfaxalone to induce its potentiating effect [7, 58].

### Alfaxalone has no long‐term impact on GABA_A_‐R current properties

The long‐term impact of whole‐animal alfaxalone sedation on GABA_A_‐R current electrophysiological properties was explored in brain tissue from animals sedated with alfaxalone and then following a series of *in vitro* tissue washes every 30 min. Our results indicate that 3 h after whole‐animal alfaxalone sedation, was sufficient to reverse any significant increase in GABA_A_‐R current decay time, peak amplitude, and area under the curve, induced by alfaxalone. Additionally, no significant difference in holding current values was found between naïve tissue and all timepoints following whole‐animal alfaxalone sedation. In the western painted turtle, unpublished results by Suganthan *et al*. showed a similar, rapid washout of alfaxalone following whole‐animal sedation. Replacing the aCSF housing the tissue slices every 30 min for 3 h was sufficient to reverse the impact of whole‐animal alfaxalone sedation on GABA_A_‐R current electrophysiological properties. Additionally, these results further demonstrate that both the acute application of alfaxalone onto naïve tissue and whole‐animal alfaxalone sedation result in no significant impact on extra‐synaptic GABA_A_‐R mediated tonic‐currents.

The potentiation of GABA_A_‐R currents in tissue from whole‐animal alfaxalone sedation 2 h was similar to values following the acute application of alfaxalone onto naïve tissue. GABA_A_‐R current decay time and area under the curve both increased but peak amplitude was found to increase only following alfaxalone sedation. These results, in combination, support previous research which identified that alfaxalone potentiates GABA‐induced activation of the GABA_A_‐R channel [6, 77, 83, 84].

Overall, our findings suggest that alfaxalone is an anesthetic compatible with electrophysiological experimentation in brain tissue following 3 h of *in vitro* washing after whole‐animal alfaxalone sedation. While our results support the reversal of the potentiated GABA‐induced Cl^−^ current response, a limitation of our study involves the analysis of any long‐term impact on general cell structure or function induced by whole‐animal alfaxalone sedation. As stated previously, alfaxalone induces structural changes upon allosteric binding to the GABA_A_‐R [3] and increases membrane fluidity at high extracellular concentrations [82]. It is therefore possible that the anesthetic may alter cellular properties in ways which remain undetected utilizing our current GABA_A_‐R‐focused methodology. Furthermore, alfaxalone is a synthetic analogue to the compound – Allopregnanolone, which contributes to increased neurogenesis, neuroplasticity, and neuroprotection [85, 86]. Both compounds are capable of activating human pregnane X receptors (h‐PXR) in the brain, and alfaxalone anesthesia induces similar effects as Allopregnanolone, including the secretion of mature brain‐derived neurotrophic factor and subsequently inducing neuroprotection [87]. PXR expression is highly conserved and plays an important role in head kidney‐mediated detoxification within rainbow trout, therefore it is possible that PXR is expressed in the goldfish brain [88, 89]. It is unknown whether PXR expression is significant in goldfish pyramidal neurons, and whether its activation in any region of the brain by alfaxalone could confound electrophysiological recordings. Future studies should focus on identifying the role of PXR in the goldfish brain, and whether its activation could cause alternative long‐ or short‐term impacts on electrophysiological recordings.

## Conclusion

Alfaxalone has been described as an anesthetic with a fast induction and fast washout in many animal models including fish. In this study, our results demonstrate that whole‐animal alfaxalone sedation has no long‐term impact on action potential properties or GABA_A_‐receptor currents. While whole‐animal alfaxalone sedation had no significant impact on action potential properties, a 3 h washout was required to reverse the alfaxalone‐mediated potentiation of GABA_A_‐R channel currents. Our results also confirm that alfaxalone induces short‐term alterations of GABA_A_‐R current electrophysiological properties, specifically at 2 h following whole‐animal alfaxalone sedation and following short‐term acute alfaxalone perfusion onto naive tissue. This study suggests that the short washout period from telencephalic brain slices associated with alfaxalone sedation, will make same‐day electrophysiological investigations in goldfish brain practical. The use of an anesthetic alongside future goldfish electrophysiological experimentation will introduce a significant improvement to fish well‐being. Furthermore, these results indicate that alfaxalone could represent an anesthetic compatible with electrophysiological studies in all fish species. Further experimentation in additional animal models will help confirm the generalizability of the rapid anesthetic washout following whole‐animal alfaxalone sedation.

## Conflict of interest

The authors declare no conflict of interest.

### Peer review

The peer review history for this article is available at https://www.webofscience.com/api/gateway/wos/peer‐review/10.1002/2211‐5463.13777.

## Author contributions

All authors contributed to conceptualization; DDS and HS contributed to data acquisition; DDS contributed to writing—original draft preparation; all authors contributed to writing—review and editing; LB contributed to supervision; DDS and LB contributed to funding acquisition. All authors have read and agreed to the published version of the manuscript.

## Supporting information


**Fig. S1.** Whole‐cell GABA_A_‐R current electrophysiological properties in naïve tissue following 15 and 30 min of control aCSF perfusion.

**Fig. S2.** Dose response relationship of alfaxalone potentiation of GABAA‐R current electrophysiological properties.

## Data Availability

The data that support the findings of this study are available from the corresponding author domenic.distefano@mail.utoronto.ca upon reasonable request.

## References

[1] Child KJ , Currie JP , Davis B , Dodds MG , Pearce DR and Twissell DJ (1971) The pharmacological properties in animals of CT1341—a new steroid anaesthetic agent. Br J Anaesth 43, 2–13. 4251319 10.1093/bja/43.1.2-a

[2] Watt JM (1975) Anaphylactic reactions after use of CT 1341 (althesin). Br Med J 3, 205–206. 10.1136/bmj.3.5977.205-a PMC1674143 1148733

[3] Chen Q , Wells MM , Arjunan P , Tillman TS , Cohen AE , Xu Y and Tang P (2018) Structural basis of neurosteroid anesthetic action on GABAA receptors. Nat Commun 9, 3972. 30266951 10.1038/s41467-018-06361-4 PMC6162318

[4] Barker JL , Harrison NL , Lange GD and Majewska MD (1986) Voltage‐clamp studies of the potentiation of Gaba‐activated chloride conductance by the steroid anesthetic alphaxalone and a reduced metabolite of progesterone in cultured rat CNS neurons. J Physiol Lond 377, P83.

[5] Cottrell GA , Lambert JJ and Peters JA (1987) Modulation of GABAA receptor activity by alphaxalone. Br J Pharmacol 90, 491–500. 3032320 10.1111/j.1476-5381.1987.tb11198.x PMC1917172

[6] Lambert JJ , Peters JA and Cottrell GA (1987) Actions of synthetic and endogenous steroids on the GABAA receptor. Trends Pharmacol Sci 8, 224–227.

[7] Cao LQ , Montana MC , Germann AL , Shin DJ , Chakrabarti S , Mennerick S , Yuede CM , Wozniak DF , Evers AS and Akk G (2018) Enhanced GABAergic actions resulting from the coapplication of the steroid 3α‐hydroxy‐5α‐pregnane‐11,20‐dione (alfaxalone) with propofol or diazepam. Sci Rep 8, 10341. 29985445 10.1038/s41598-018-28754-7 PMC6037692

[8] Chisari M , Eisenman LN , Krishnan K , Bandyopadhyaya AK , Wang C , Taylor A , Benz A , Covey DF , Zorumski CF and Mennerick S (2009) The influence of neuroactive steroid Lipophilicity on GABAA receptor modulation: evidence for a low‐affinity interaction. J Neurophysiol 102, 1254–1264. 19553485 10.1152/jn.00346.2009 PMC2724350

[9] Muir W , Lerche P , Wiese A , Nelson L , Pasloske K and Whittem T (2008) Cardiorespiratory and anesthetic effects of clinical and supraclinical doses of alfaxalone in dogs. Vet Anaesth Analg 35, 451–462. 18793290 10.1111/j.1467-2995.2008.00406.x

[10] West JA (2017) Alfaxalone. J Exot Pet Med 26, 156–161.

[11] Ferré PJ , Pasloske K , Whittem T , Ranasinghe MG , Li Q and Lefebvre HP (2006) Plasma pharmacokinetics of alfaxalone in dogs after an intravenous bolus of Alfaxan‐CD RTU. Vet Anaesth Analg 33, 229–236. 16764587 10.1111/j.1467-2995.2005.00264.x

[12] Goodwin WA , Keates HL , Pasloske K , Pearson M , Sauer B and Ranasinghe MG (2011) The pharmacokinetics and pharmacodynamics of the injectable anaesthetic alfaxalone in the horse. Vet Anaesth Analg 38, 431–438. 21831048 10.1111/j.1467-2995.2011.00634.x

[13] Goodwin W , Keates H , Pasloske K , Pearson M , Sauer B and Ranasinghe MG (2012) Plasma pharmacokinetics and pharmacodynamics of alfaxalone in neonatal foals after an intravenous bolus of alfaxalone following premedication with butorphanol tartrate. Vet Anaesth Analg 39, 503–510. 22642499 10.1111/j.1467-2995.2012.00734.x

[14] Lau C , Ranasinghe MG , Shiels I , Keates H , Pasloske K and Bellingham MC (2013) Plasma pharmacokinetics of alfaxalone after a single intraperitoneal or intravenous injection of Alfaxan® in rats. J Vet Pharmacol Ther 36, 516–520. 23600373 10.1111/jvp.12055

[15] Rodrigo‐Mocholí D , Escudero E , Belda E , Laredo F , Hernandis V and Marín P (2018) Pharmacokinetics and effects of alfaxalone after intravenous and intramuscular administration to cats. N Z Vet J 66, 172–177. 29562829 10.1080/00480169.2018.1455541

[16] Minter LJ , Bailey KM , Harms CA , Lewbart GA and Posner LP (2014) The efficacy of alfaxalone for immersion anesthesia in koi carp (*Cyprinus carpio*). Vet Anaesth Analg 41, 398–405. 24754530 10.1111/vaa.12113

[17] Bugman AM , Langer PT , Hadzima E , Rivas AE and Mitchell MA (2016) Evaluation of the anesthetic efficacy of alfaxalone in oscar fish (*Astronotus ocellatus*). Am J Vet Res 77, 239–244. 26919593 10.2460/ajvr.77.3.239

[18] Leonardi F , Costa GL , Interlandi CD , Rosa J , Ghidelli A and Musicò M (2019) Immersion anaesthesia in goldfish (*Carassius auratus*) with three concentrations of alfaxalone. Vet Anaesth Analg 46, 79–83. 30528215 10.1016/j.vaa.2018.08.006

[19] Farry T , Lau C , Keates H , Pasloske K , Woldeyohannes S , Allavena R and Goodwin W (2022) Comparison of two formulations of alfaxalone for immersion anaesthesia in laboratory zebrafish (*Danio rerio*). Vet Anaesth Analg 49, 473–476. 35718716 10.1016/j.vaa.2022.05.001

[20] Savson DJ , Zenilman SS , Smith CR , Daugherity EK , Singh B and Getchell RG (2022) Comparison of alfaxalone and tricaine methanesulfonate immersion anesthesia and alfaxalone residue clearance in rainbow trout (*Oncorhynchus mykiss*). Comp Med 72, 181–194. 35659382 10.30802/AALAS-CM-22-000052 PMC9334004

[21] Neiffer DL and Stamper MA (2009) Fish sedation, anesthesia, analgesia, and euthanasia: considerations, methods, and types of drugs. ILAR J 50, 343–360. 19949251 10.1093/ilar.50.4.343

[22] Zahl IH , Samuelsen O and Kiessling A (2012) Anaesthesia of farmed fish: implications for welfare. Fish Physiol Biochem 38, 201–218. 22160749 10.1007/s10695-011-9565-1

[23] Canadian Council on Animal Care (2021) CCAC animal data report .

[24] Sollid J , De Angelis P , Gundersen K and Nilsson GE (2003) Hypoxia induces adaptive and reversible gross morphological changes in crucian carp gills. J Exp Biol 206, 3667–3673. 12966058 10.1242/jeb.00594

[25] Bickler PE and Buck LT (2007) Hypoxia tolerance in reptiles, amphibians, and fishes: life with variable oxygen availability. Annu Rev Physiol 69, 145–170. 17037980 10.1146/annurev.physiol.69.031905.162529

[26] Piironen J and Holopainen IJ (1986) A note on seasonality in anoxia tolerance of crucian carp (*Carassius carassius* (L.)) in the laboratory. Ann Zool Fenn 23, 335–338.

[27] Rausch RN , Crawshaw LI and Wallace HL (2000) Effects of hypoxia, anoxia, and endogenous ethanol on thermoregulation in goldfish, *Carassius auratus* . Am J Physiol Regul Integr Comp Physiol 278, R545–R555. 10712271 10.1152/ajpregu.2000.278.3.R545

[28] Lee J‐M , Grabb MC , Zipfel GJ and Choi DW (2000) Brain tissue responses to ischemia. J Clin Invest 106, 723–731. 10995780 10.1172/JCI11003 PMC381398

[29] Hochachka PW and Somero GN (1984) Biochemical Adaptation, Vol. 538. Princeton University Press, Princeton, NJ.

[30] Hochachka PW (1986) Metabolic arrest. Intensive Care Med 12, 127–133. 2426320 10.1007/BF00254926

[31] Van Waversveld J , Addink ADF and Van Den Thillart G (1989) Simultaneous direct and indirect calorimetry on normoxic and anoxic goldfish. J Exp Biol 142, 325–335.

[32] Lutz PL and Nilsson GE (2004) Vertebrate brains at the pilot light. Respir Physiol Neurobiol 141, 285–296. 15288600 10.1016/j.resp.2004.03.013

[33] Hochachka PW (1986) Defense strategies against hypoxia and hypothermia. Science 231, 234–241. 2417316 10.1126/science.2417316

[34] Diemer NH , Jørgensen MB , Johansen FF , Sheardown M and Honoré T (1992) Protection against ischemic hippocampal CAI damage in the rat with a new non‐NMDA antagonist, NBQX. Acta Neurol Scand 86, 45–49. 1325729 10.1111/j.1600-0404.1992.tb08052.x

[35] Wilkie MP , Pamenter ME , Alkabie S , Carapic D , Shin DSH and Buck LT (2008) Evidence of anoxia‐induced channel arrest in the brain of the goldfish (*Carassius auratus*). Comp Biochem Physiol C Toxicol Pharmacol 148, 355–362. 18620076 10.1016/j.cbpc.2008.06.004

[36] Nilsson GE and Lutz PL (1992) Adenosine release in the anoxic turtle brain: a possible mechanism for anoxic survival. J Exp Biol 162, 345–351.

[37] Pamenter ME , Hogg DW , Ormond J , Shin DS , Woodin MA and Buck LT (2011) Endogenous GABAA and GABAB receptor‐mediated electrical suppression is critical to neuronal anoxia tolerance. Proc Natl Acad Sci USA 108, 11274–11279. 21690381 10.1073/pnas.1102429108 PMC3131309

[38] Buck LT and Pamenter ME (2018) The hypoxia‐tolerant vertebrate brain: arresting synaptic activity. Comp Biochem Physiol B Biochem Mol Biol 224, 61–70. 29223874 10.1016/j.cbpb.2017.11.015

[39] Ashley PJ , Sneddon LU and McCrohan CR (2007) Nociception in fish: stimulus–response properties of receptors on the head of trout *Oncorhynchus mykiss* . Brain Res 1166, 47–54. 17673186 10.1016/j.brainres.2007.07.011

[40] Roques JAC , Abbink W , Geurds F , van de Vis H and Flik G (2010) Tailfin clipping, a painful procedure: studies on *Nile tilapia* and common carp. Physiol Behav 101, 533–540. 20705079 10.1016/j.physbeh.2010.08.001

[41] Sneddon LU (2012) Clinical anesthesia and analgesia in fish. J Exot Pet Med 21, 32–43.

[42] Braithwaite V (2010) Do Fish Feel Pain? Oxford University Press, Oxford, UK.

[43] Rose JD , Arlinghaus R , Cooke SJ , Diggles BK , Sawynok W , Stevens ED and Wynne CDL (2014) Can fish really feel pain? Fish Fish 15, 97–133.

[44] Chatigny F , Creighton CM and Stevens ED (2018) Updated review of fish analgesia. J Am Assoc Lab Anim Sci 57, 5–12. 29402345 PMC5875091

[45] Neumcke B , Schwarz W and Stämpfli R (1981) Block of Na channels in the membrane of myelinated nerve by benzocaine. Pflugers Arch 390, 230–236. 6265861 10.1007/BF00658267

[46] Carter KM , Woodley CM and Brown RS (2011) A review of tricaine methanesulfonate for anesthesia of fish. Rev Fish Biol Fish 21, 51–59.

[47] Hayton WL , Szoke A , Kemmenoe BH and Vick AM (1996) Disposition of benzocaine in channel catfish. Aquat Toxicol 36, 99–113.

[48] Aoshima H and Hamamoto K (1999) Potentiation of GABA a receptors expressed in *Xenopus* oocytes by perfume and phytoncid. Biosci Biotechnol Biochem 63, 743–748. 10361687 10.1271/bbb.63.743

[49] Lee MH , Yeon K‐Y , Park C‐K , Li H‐Y , Fang Z , Kim MS , Choi S‐Y , Lee SJ , Lee S , Park K *et al*. (2005) Eugenol inhibits calcium currents in dental afferent neurons. J Dent Res 84, 848–851. 16109996 10.1177/154405910508400913

[50] Park C‐K , Li HY , Yeon K‐Y , Jung SJ , Choi S‐Y , Lee SJ , Lee S , Park K , Kim JS and Oh SB (2006) Eugenol inhibits sodium currents in dental afferent neurons. J Dent Res 85, 900–904. 16998128 10.1177/154405910608501005

[51] Li HY , Park C‐K , Jung SJ , Choi S‐Y , Lee SJ , Park K , Kim JS and Oh SB (2007) Eugenol inhibits K^+^ currents in trigeminal ganglion neurons. J Dent Res 86, 898–902. 17720863 10.1177/154405910708600918

[52] Guénette SA , Uhland FC , Hélie P , Beaudry F and Vachon P (2007) Pharmacokinetics of eugenol in rainbow trout (*Oncorhynchus mykiss*). Aquaculture 266, 262–265.

[53] Hill JV and Forster ME (2004) Cardiovascular responses of Chinook salmon (*Oncorhynchus tshawytscha*) during rapid anaesthetic induction and recovery. Comp Biochem Physiol C Toxicol Pharmacol 137, 167–177. 15050928 10.1016/j.cca.2004.01.002

[54] Hossein‐Javaheri N , Wilkie MP , Lado WE and Buck LT (2017) Stellate and pyramidal neurons in goldfish telencephalon respond differently to anoxia and GABA receptor inhibition. J Exp Biol 220, 695–704. 27923876 10.1242/jeb.146605

[55] Blanton MG , Lo Turco JJ and Kriegstein AR (1989) Whole cell recording from neurons in slices of reptilian and mammalian cerebral cortex. J Neurosci Methods 30, 203–210. 2607782 10.1016/0165-0270(89)90131-3

[56] Hawrysh PJ and Buck LT (2019) Mitochondrial matrix pH acidifies during anoxia and is maintained by the F1Fo‐ATPase in anoxia‐tolerant painted turtle cortical neurons. FEBS Open Bio 9, 571–581. 10.1002/2211-5463.12612 PMC6443863 30984533

[57] Connors BW and Kriegstein AR (1986) Cellular physiology of the turtle visual cortex: distinctive properties of pyramidal and stellate neurons. J Neurosci 6, 164–177. 3944618 10.1523/JNEUROSCI.06-01-00164.1986 PMC6568632

[58] Miles AR , Hawrysh PJ , Hossein‐Javaheri N and Buck LT (2018) Taurine activates glycine and GABAA receptor currents in anoxia‐tolerant painted turtle pyramidal neurons. J Exp Biol 221, jeb181529. 30237241 10.1242/jeb.181529

[59] Moldavan M , Cravetchi O and Allen CN (2021) Diurnal properties of tonic and synaptic GABAA receptor‐mediated currents in suprachiasmatic nucleus neurons. J Neurophysiol 126, 637–652. 34259044 10.1152/jn.00556.2020 PMC8409954

[60] Pillai V , Buck L and Lari E (2021) Scavenging of reactive oxygen species mimics the anoxic response in goldfish pyramidal neurons. J Exp Biol 224, jeb238147. 34047778 10.1242/jeb.238147

[61] Baranauskas G (2007) Ionic channel function in action potential generation: current perspective. Mol Neurobiol 35, 129–150. 17917103 10.1007/s12035-007-8001-0

[62] Rao VR and Finkbeiner S (2007) NMDA and AMPA receptors: old channels, new tricks. Trends Neurosci 30, 284–291. 17418904 10.1016/j.tins.2007.03.012

[63] Lavoie AM , Tingey JJ , Harrison NL , Pritchett DB and Twyman RE (1997) Activation and deactivation rates of recombinant GABA(a) receptor channels are dependent on alpha‐subunit isoform. Biophys J 73, 2518–2526. 9370445 10.1016/S0006-3495(97)78280-8 PMC1181153

[64] Sallard E , Letourneur D and Legendre P (2021) Electrophysiology of ionotropic GABA receptors. Cell Mol Life Sci 78, 5341–5370. 34061215 10.1007/s00018-021-03846-2 PMC8257536

[65] Jones MV and Westbrook GL (1995) Desensitized states prolong GABAA channel responses to brief agonist pulses. Neuron 15, 181–191. 7542462 10.1016/0896-6273(95)90075-6

[66] Chang Y , Ghansah E , Chen Y , Ye J and Weiss DS (2002) Desensitization mechanism of GABA receptors revealed by single oocyte binding and receptor function. J Neurosci 22, 7982–7990. 12223551 10.1523/JNEUROSCI.22-18-07982.2002 PMC6758118

[67] Overstreet LS , Jones MV and Westbrook GL (2000) Slow desensitization regulates the availability of synaptic GABAA receptors. J Neurosci 20, 7914–7921. 11050111 10.1523/JNEUROSCI.20-21-07914.2000 PMC6772729

[68] Rossi DJ and Hamann M (1998) Spillover‐mediated transmission at inhibitory synapses promoted by high affinity α6 subunit GABAA receptors and glomerular geometry. Neuron 20, 783–795. 9581769 10.1016/s0896-6273(00)81016-8

[69] Farrant M and Nusser Z (2005) Variations on an inhibitory theme: phasic and tonic activation of GABAA receptors. Nat Rev Neurosci 6, 215–229. 15738957 10.1038/nrn1625

[70] Kaneda M , Farrant M and Cull‐Candy SG (1995) Whole‐cell and single‐channel currents activated by GABA and glycine in granule cells of the rat cerebellum. J Physiol 485, 419–435. 7545231 10.1113/jphysiol.1995.sp020739 PMC1158002

[71] Ben‐Ari Y , Gaiarsa J‐L , Tyzio R and Khazipov R (2007) GABA: a Pioneer transmitter that excites immature neurons and generates primitive oscillations. Physiol Rev 87, 1215–1284. 17928584 10.1152/physrev.00017.2006

[72] Simon J , Wakimoto H , Fujita N , Lalande M and Barnard EA (2004) Analysis of the set of GABAA receptor genes in the human genome. J Biol Chem 279, 41422–41435. 15258161 10.1074/jbc.M401354200

[73] Sieghart W (2000) Unraveling the function of GABAA receptor subtypes. Trends Pharmacol Sci 21, 411–413. 11121567 10.1016/s0165-6147(00)01564-9

[74] Brickley SG , Cull‐Candy SG and Farrant M (1999) Single‐channel properties of synaptic and extrasynaptic GABAA receptors suggest differential targeting of receptor subtypes. J Neurosci 19, 2960–2973. 10191314 10.1523/JNEUROSCI.19-08-02960.1999 PMC6782265

[75] Maitra R and Reynolds JN (1998) Modulation of GABAA receptor function by neuroactive steroids: evidence for heterogeneity of steroid sensitivity of recombinant GABAA receptor isoforms. Can J Physiol Pharmacol 76, 909–920. 10066142 10.1139/cjpp-76-9-909

[76] Lau C , Thakre PP and Bellingham MC (2019) Alfaxalone causes reduction of glycinergic IPSCs, but not glutamatergic EPSCs, and activates a depolarizing current in rat hypoglossal motor neurons. Front Cell Neurosci 13, 100. 30967762 10.3389/fncel.2019.00100 PMC6440435

[77] Barker JL , Harrison NL , Lange GD and Owen DG (1987) Potentiation of gamma‐aminobutyric‐acid‐activated chloride conductance by a steroid anaesthetic in cultured rat spinal neurones. J Physiol 386, 485–501. 2445967 10.1113/jphysiol.1987.sp016547 PMC1192475

[78] Harrison NL , Vicini S and Barker JL (1987) A steroid anesthetic prolongs inhibitory postsynaptic currents in cultured rat hippocampal neurons. J Neurosci 7, 604–609. 3819824 10.1523/JNEUROSCI.07-02-00604.1987 PMC6568915

[79] Bai D , Zhu G , Pennefather P , Jackson MF , MacDonald JF and Orser BA (2001) Distinct functional and pharmacological properties of tonic and quantal inhibitory postsynaptic currents mediated by γ‐aminobutyric AcidA receptors in hippocampal neurons. Mol Pharmacol 59, 814–824. 11259626 10.1124/mol.59.4.814

[80] Banks MI and Pearce RA (2000) Kinetic differences between synaptic and Extrasynaptic GABAA receptors in CA1 pyramidal cells. J Neurosci 20, 937–948. 10648698 10.1523/JNEUROSCI.20-03-00937.2000 PMC6774173

[81] Hogg DW , Pamenter ME , Dukoff DJ and Buck LT (2015) Decreases in mitochondrial reactive oxygen species initiate GABAA receptor‐mediated electrical suppression in anoxia‐tolerant turtle neurons. J Physiol 593, 2311–2326. 25781154 10.1113/JP270474 PMC4457194

[82] Lawrence DK and Gill EW (1975) Structurally specific effects of some steroid anesthetics on spin‐labeled liposomes. Mol Pharmacol 11, 280–286. 167275

[83] Lambert JJ , Belelli D , Peden DR , Vardy AW and Peters JA (2003) Neurosteroid modulation of GABAA receptors. Prog Neurobiol 71, 67–80. 14611869 10.1016/j.pneurobio.2003.09.001

[84] Warne LN , Beths T , Whittem T , Carter JE and Bauquier SH (2015) A review of the pharmacology and clinical application of alfaxalone in cats. Vet J 203, 141–148. 25582797 10.1016/j.tvjl.2014.12.011

[85] Langmade SJ , Gale SE , Frolov A , Mohri I , Suzuki K , Mellon SH , Walkley SU , Covey DF , Schaffer JE and Ory DS (2006) Pregnane X receptor (PXR) activation: a mechanism for neuroprotection in a mouse model of Niemann–pick C disease. Proc Natl Acad Sci USA 103, 13807–13812. 16940355 10.1073/pnas.0606218103 PMC1564205

[86] Singh C , Liu L , Wang JM , Irwin RW , Yao J , Chen S , Henry S , Thompson RF and Brinton RD (2012) Allopregnanolone restores hippocampal‐dependent learning and memory and neural progenitor survival in aging 3xTgAD and nonTg mice. Neurobiol Aging 33, 1493–1506. 21803451 10.1016/j.neurobiolaging.2011.06.008 PMC3232295

[87] Serrao JM and Goodchild CS (2022) Alfaxalone anaesthesia increases brain derived neurotrophic factor levels and preserves postoperative cognition by activating pregnane‐X receptors: an in vitro study and a double blind randomised controlled trial. BMC Anesthesiol 22, 401. 36564723 10.1186/s12871-022-01940-x PMC9789577

[88] Frye CA , Koonce CJ and Walf AA (2014) Involvement of pregnane xenobiotic receptor in mating‐induced allopregnanolone formation in the midbrain and hippocampus and brain‐derived neurotrophic factor in the hippocampus among female rats. Psychopharmacology 231, 3375–3390. 24781516 10.1007/s00213-014-3569-3 PMC4135012

[89] Liu C , Wang B , Zhou B , Jian X , Zhang X and Wang Y (2019) The responses of *Oncorhynchus mykiss* coping with BDE‐47 stress via PXR‐mediated detoxification and Nrf2‐mediated antioxidation system. Aquat Toxicol 207, 63–71. 30530205 10.1016/j.aquatox.2018.11.026

