# Supplementary material for: Alfaxalone does not have long‐term effects on goldfish pyramidal neuron action potential properties or GABAA receptor currents
Source: FEBS Open Bio. 2024 Feb 11;14(4):555–73. doi: 10.1002/2211-5463.13777 (PMC10988724; doi:10.1002/2211-5463.13777)
Supplement: Supplementary file 1 — Fig. S1. Whole‐cell GABAA‐R current electrophysiological properties in naïve tissue following 15 and 30 min of control aCSF perfusion. [file FEB4-14-555-s001.docx]

**
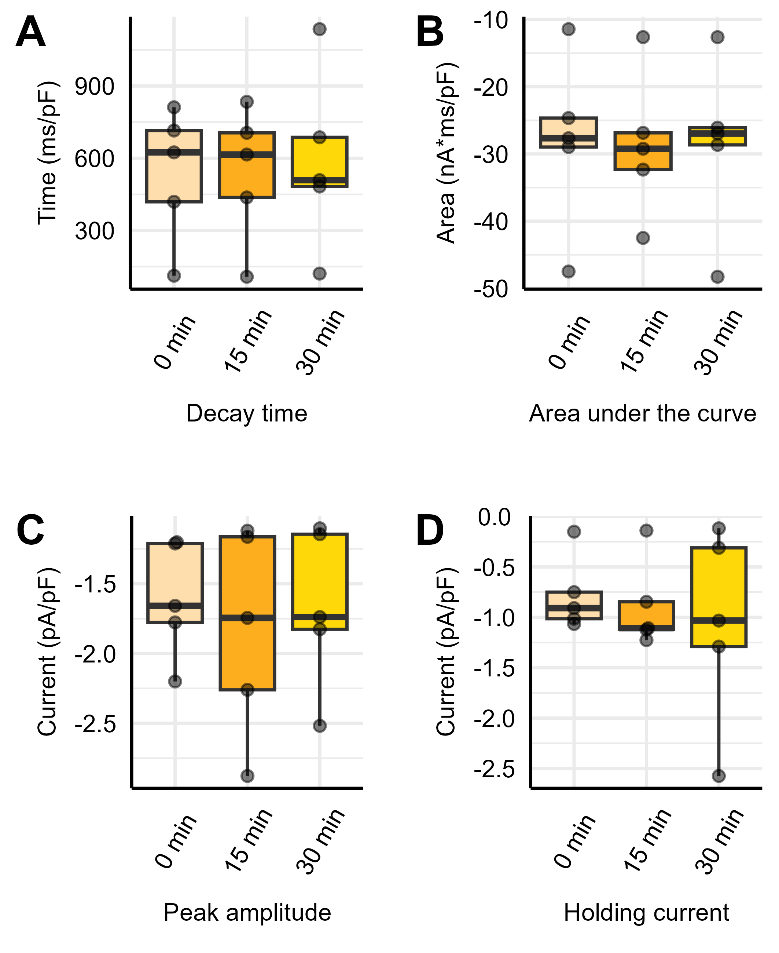
**

**Sup. 1. Whole-cell GABA_A_-R current electrophysiological properties in naïve tissue following 15 and 30 min of control aCSF perfusion.** Properties include (A) decay time, (B) area under the curve, (C) peak amplitude, and (D) passive holding current. No statistically significant difference was found between the three time points for any of the electrophysiological properties, including decay time (*P* = 0.551, *n* = 5), area under the curve (*P* = 0.917, *n* = 5), peak amplitude (*P* = 0.218, *n* = 5), and passive holding current (*P* = 0.540, *n* = 5). GABA_A_-R currents were elicited through clamping the cell voltage at -80 mV and perfusing 2 mM GABA onto the tissue slice for 1-2 seconds. This process was repeated after 15 minutes, and 30 minutes using the same whole-cell patch to confirm long-term stability of the whole-cell patch and GABA_A_-R current. Each point represents data from a separate experiment (n = 5). Statistical significance determined using a repeated measures ANOVA.
