# Supplementary material for: Alfaxalone does not have long‐term effects on goldfish pyramidal neuron action potential properties or GABAA receptor currents
Source: FEBS Open Bio. 2024 Feb 11;14(4):555–73. doi: 10.1002/2211-5463.13777 (PMC10988724; doi:10.1002/2211-5463.13777)
Supplement: Supplementary file 2 — Fig. S2. Dose response relationship of alfaxalone potentiation of GABAA‐R current electrophysiological properties. [file FEB4-14-555-s002.docx]

**
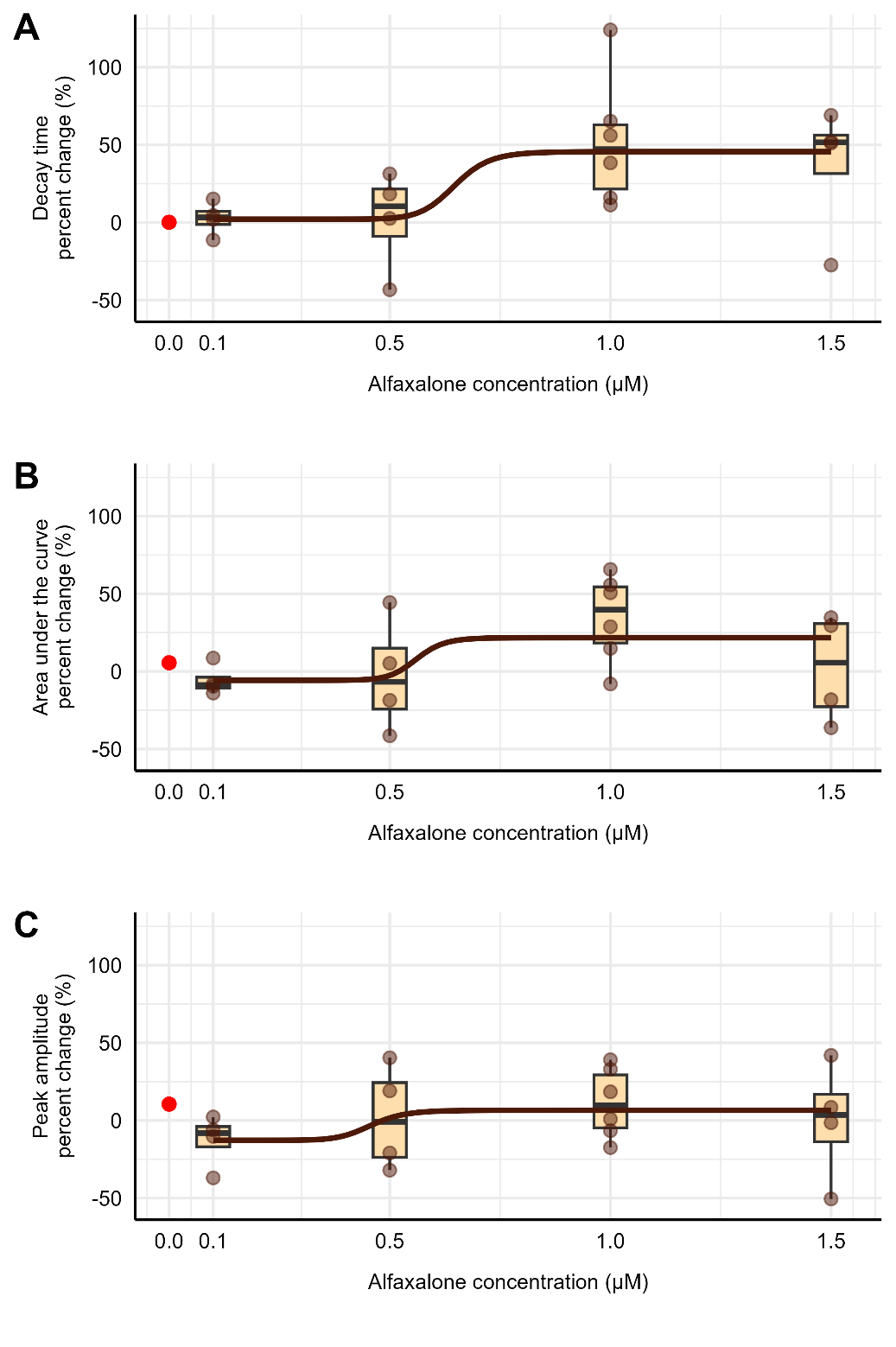
**

**Sup. 2. Dose response relationship of alfaxalone potentiation of GABAA-R current electrophysiological properties.** Properties include the percent change in (A) decay time, (B) area under the curve, and (C) peak amplitude following acute alfaxalone application (0.1-1.5 µM) relative to control GABA_A_-R decay time, fit to the four-parameter Hill equation (log logistic equation). The red point represents the average percent change in each property value following 15 minutes of control aCSF perfusion. Minimal peak amplitude potentiation occurred at increasing concentrations of alfaxalone resulting in a poor fit to the Hill equation. Alfaxalone concentrations greater than 1.5 µM resulted in patch destabilization within 10 minutes, and patch failure. The alfaxalone-induced potentiation of all properties saturated at approximately 1 µM, with minimal patch destabilization. Therefore, it was determined that the acute application of 1 µM alfaxalone onto naïve tissue wound be utilized for the remainder of the study. GABA_A_-R currents were elicited through clamping the cell voltage at -80 mV and perfusing 2 mM GABA onto the tissue slice for 1-2 seconds. This process was repeated after 15 minutes of 0.1-1.5 µM alfaxalone perfusion. Percent change responses were expressed relative to the response in the absence of alfaxalone using the equation [(A-C)/C]*100%, where A represents GABA_A_-R current property measurements following acute alfaxalone treatment and C represents GABA_A_-R current property measurements under control conditions [8]. Each point represents data from a separate experiment (n = 4-6).
